# Supplementary material for: Constitution of Long COVID illness, patienthood and recovery: a critical synthesis of qualitative studies
Source: BMJ Open. 2024 Mar 28;14(3):e083340. doi: 10.1136/bmjopen-2023-083340 (PMC10982801; doi:10.1136/bmjopen-2023-083340)
Supplement: Supplementary data [file bmjopen-2023-083340supp001.pdf]

BMJ Publishing Group Limited (BMJ) disclaims all liability and responsibility arising from any reliance placed on this supplemental material which has been supplied by the author(s)

Supplemental Table 1: Database search queries

Database	Search Query	Results
PubMed	((postacute[Title/Abstract]) OR ("post acute"[Title/Abstract]) OR (pasc[Title/Abstract]) OR (persistent symptoms[Title/Abstract]) OR (prolonged symptoms[Title/Abstract]) OR ("long covid"[Title/Abstract]) OR (post covid syndrome[Title/Abstract])) AND (qualitative[Title/Abstract]) AND (covid[Title/Abstract])	144
Web of Science	(AB=(postacute) OR AB=("post acute") OR AB=(pasc) OR AB=(persistent symptoms) OR AB=(prolonged symptoms) OR AB=("long covid") OR AB=(post covid syndrome)) AND AB=(qualitative) AND AB=(covid)	187
