# Supplementary material for: Constitution of Long COVID illness, patienthood and recovery: a critical synthesis of qualitative studies
Source: BMJ Open. 2024 Mar 28;14(3):e083340. doi: 10.1136/bmjopen-2023-083340 (PMC10982801; doi:10.1136/bmjopen-2023-083340)
Supplement: Supplementary data [file bmjopen-2023-083340supp002.pdf]

BMJ Publishing Group Limited (BMJ) disclaims all liability and responsibility arising from any reliance placed on this supplemental material which has been supplied by the author(s)

Supplemental Table 2: Quality appraisal prompts

- Are the objectives of the research clearly stated?
- Is the research design clearly described and appropriate?
- Are data collection methods transparent?
- Are data analysis methods clearly described and appropriate?
- Are there sufficient data to support the authors' interpretations?

Quality appraisal prompts citation:

Harrison M, Rhodes T, Lancaster K. How do care environments shape healthcare? A synthesis of qualitative studies among healthcare workers during the COVID-19 pandemic. *BMJ Open*. 2022;12(9):e063867. DOI: 10.1136/bmjopen-2022-063867
