# Supplementary material for: Constitution of Long COVID illness, patienthood and recovery: a critical synthesis of qualitative studies
Source: BMJ Open. 2024 Mar 28;14(3):e083340. doi: 10.1136/bmjopen-2023-083340 (PMC10982801; doi:10.1136/bmjopen-2023-083340)
Supplement: Author's manuscript [file bmjopen-2023-083340.draft_revisions.pdf]

BMJ Open

BMJ Open is committed to open peer review. As part of this commitment we make the peer review history of every article we publish publicly available.

When an article is published we post the peer reviewers' comments and the authors' responses online. We also post the versions of the paper that were used during peer review. These are the versions that the peer review comments apply to.

The versions of the paper that follow are the versions that were submitted during the peer review process. They are not the versions of record or the final published versions. They should not be cited or distributed as the published version of this manuscript.

BMJ Open is an open access journal and the full, final, typeset and author-corrected version of record of the manuscript is available on our site with no access controls, subscription charges or pay-per-view fees (http://bmjopen.bmj.com).

If you have any questions on BMJ Open's open peer review process please email info.bmjopen@bmj.com

BMJ Open

The constitution of Long Covid illness, patienthood, and recovery: A critical synthesis of qualitative studies

Journal:	*BMJ Open*
Manuscript ID	bmjopen-2023-083340
Article Type:	Original research
Date Submitted by the Author:	19-Dec-2023
Complete List of Authors:	Harrison, Mia; University of New South Wales, Centre for Social Research in Health<br>Rhodes, Tim; London School of Hygiene & Tropical Medicine; University of New South Wales, Centre for Social Research in Health<br>Lancaster, Kari; University of New South Wales, Centre for Social Research in Health; Goldsmiths University of London,
Keywords:	Post-Acute COVID-19 Syndrome, Patients, QUALITATIVE RESEARCH, COVID-19, Patient-Centered Care

SCHOLARONE™
Manuscripts

BMJ

I, the Submitting Author has the right to grant and does grant on behalf of all authors of the Work (as defined in the below author licence), an exclusive licence and/or a non-exclusive licence for contributions from authors who are: i) UK Crown employees; ii) where BMJ has agreed a CC-BY licence shall apply, and/or iii) in accordance with the terms applicable for US Federal Government officers or employees acting as part of their official duties; on a worldwide, perpetual, irrevocable, royalty-free basis to BMJ Publishing Group Ltd ("BMJ") its licensees and where the relevant Journal is co-owned by BMJ to the co-owners of the Journal, to publish the Work in this journal and any other BMJ products and to exploit all rights, as set out in our licence.

The Submitting Author accepts and understands that any supply made under these terms is made by BMJ to the Submitting Author unless you are acting as an employee on behalf of your employer or a postgraduate student of an affiliated institution which is paying any applicable article publishing charge ("APC") for Open Access articles. Where the Submitting Author wishes to make the Work available on an Open Access basis (and intends to pay the relevant APC), the terms of reuse of such Open Access shall be governed by a Creative Commons licence – details of these licences and which Creative Commons licence will apply to this Work are set out in our licence referred to above.

Other than as permitted in any relevant BMJ Author's Self Archiving Policies, I confirm this Work has not been accepted for publication elsewhere, is not being considered for publication elsewhere and does not duplicate material already published. I confirm all authors consent to publication of this Work and authorise the granting of this licence.

For peer review only

Title

The constitution of Long Covid illness, patienthood, and recovery: A critical synthesis of qualitative studies

Corresponding author

Mia Harrison

Centre for Social Research in Health, University of New South Wales, High St, Kensington, NSW 2052, Australia.

mia.harrison@unsw.edu.au

Author 1

Mia Harrison

1) Centre for Social Research in Health, University of New South Wales, Sydney, Australia

Author 2

Tim Rhodes

1) London School of Hygiene & Tropical Medicine, London, United Kingdom
2) Centre for Social Research in Health, University of New South Wales, Sydney, Australia

Author 3

Kari Lancaster

1) Centre for Social Research in Health, University of New South Wales, Sydney, Australia
2) Goldsmiths, University of London

Word Count

5073

For peer review only

Abstract

Objective: To investigate lived experiences of Long Covid.

Design: Critical interpretive synthesis (CIS) of qualitative research.

Participants: Studies included qualitative research investigating experiences of Long Covid.

Results: 68 articles were identified in the first phase of sampling with 16 studies and 879 participants included in the final synthesis. Analysis of these studies was organised into three thematic constructions of Long Covid: 1) the illness; 2) the patient; 3) recovery. Long Covid was diversely characterised across study introductions, designs, and findings, but was underpinned by shared diagnostic logics, which emphasise the identification and measurement of symptoms. The boundaries between different constitutions of Long Covid were often imprecise, and slippages between different definitions of Long Covid had implications for patient experiences of seeking a diagnosis, receipt of formal and informal care, and expectations of recovery.

Conclusions: Long Covid is a site of multiple and diverse qualitative interpretation. Accounts of lived experience emphasise constitutions of illness, patienthood, and recovery as situated and emergent. The ongoing context-based negotiation of Long Covid is a defining qualitative feature of the condition. Approaches to researching, diagnosing, and developing health interventions must be as adaptive as the varieties of Long Covid lived experience.

Keywords

Qualitative synthesis; Long Covid; Lived experience; Patienthood; Recovery

Article Summary

Strengths and limitations of this study

- This study employed a critical interpretive synthesis method for review of literature, which enabled the development of new insights about the constitution of Long Covid, extending the goals of the included studies.
- This study developed synthetic constructs that allowed for analysis of primary evidence and second order analyses, which enabled the evaluation of how the methods and approaches of included studies shaped how Long Covid could be understood.
- Included studies primarily recruited from Long Covid support groups or were developed as qualitative adjuncts to existing Long Covid cohort studies.
- Included studies sampled primarily from UK and US contexts.
- Included studies primarily used interview and focus group methods.

For peer review only - http://bmjopen.bmj.com/site/about/guidelines.xhtml

Introduction

This paper synthesises qualitative research into lived experiences of Long Covid. Long Covid is generally described as an uncertain, emerging, and complex illness or health condition, consisting of a broad range of possible symptoms that persist for a minimum of between 3 and 12 weeks following Covid-19 infection [1-3]. As research and awareness of the long-term health effects of Covid-19 have increased, understandings of Long Covid have broadened to encompass diverse illness effects, care needs, and trajectories of illness and recovery [4, 5]. This has led to the rapid development of clinical knowledge and practice guidelines (coproduced via clinician and patient collaboration) that account for variations and complex presentations of Long Covid [6]. Yet the classification of Long Covid and expectations of post-Covid-19 recovery remain a subject of contestation and social and medical critique [4, 5, 7].

Systematic reviews of Long Covid studies have investigated characteristics, frequency, and heterogeneity of individual Long Covid symptoms (e.g., fatigue, cough, chest pain, memory loss, dyspnea, depression) [1, 8-10]. These reviews also noted temporal information such as time of follow up or average symptom duration, with one study systematically mapping the temporal persistence of individual symptoms post-infection at 12 weeks and 6 months [10]. Other reviews have synthesised studies focused on specific symptoms into categories, such as affected bodily systems (e.g., cardiopulmonary, respiratory, multisystem) [11-13] or areas of health (i.e., physical, mental, social) [14]. One review specifically compared clinical presentation and symptomology of Long Covid and myalgic encephalomyelitis/chronic fatigue syndrome (ME/CFS) [15].

Reviews have also investigated Long Covid interventions, management, and implications for clinical practice [2, 12, 16, 17]. One review grouped post-Covid symptoms into proposed subtypes (e.g., non-severe Covid-19 multi-organ sequelae, pulmonary fibrosis sequelae, ME/CFS) and identified potential interventions for each subtype [18].

Previous review work has tended to focus on clinical constitutions of Long Covid, with inadequate attention to how Long Covid is experienced and shaped by social relations and contexts. Reviews focusing on qualitative evidence have greater contributed to such areas, though these are limited. One qualitative review of 4 studies focused on the specific experiences of living with pulmonary sequalae and its implications for nursing practice [2]. Other reviews of qualitative research on living with Long Covid have synthesised the emotional and psychosocial impacts of Long Covid and related experiences in accessing healthcare and resources [3, 19, 20].

One scoping review took a critical sociological approach, synthesising 93 studies (including qualitative studies) to explore clinical definitions and diagnostic constructions of Long Covid [5]. This review argued that research efforts to reduce medical uncertainty by broadening or solidifying the conceptual boundaries of ‘Long Covid’ have produced other forms ignorance in medical knowledge and clinical care [5]. This contributes to an emerging body of critical literature investigating how Long Covid has come to be classified across medical and social domains [4, 7, 21].

For peer review only - http://bmjopen.bmj.com/site/about/guidelines.xhtml

This paper responds to and extends upon critiques regarding fluctuating classifications of Long Covid and their implications for practice and policy [4, 5, 7, 21]. We develop a critical interpretive synthesis of qualitative research on experiences of Long Covid, attending to both qualitative accounts of these experiences and how they become understood through the research. Following Mol and Hardon [22], we approach the constitution of Long Covid as differently made in relation with emergent knowledges, including those of science and community clinicians, and among clinicians, patients and other stakeholders. Making sense of Long Covid is thus "a matter […] of negotiation," in which we eschew any goal of a single and definitive description of Long Covid, and instead develop tools for understanding the experiences, stakes, and implications of Long Covid in different health and social situations, and for patients themselves [22]. Our analysis brings emerging clinical understandings of Long Covid as an illness or diagnostic category into dialogue with experiences of patient care and recovery. This enables us to attend to Long Covid in relation to the illness, the patient, and recovery experience as separate but interconnected concerns that generate diverse yet practical insights.

Methodology

This paper uses critical interpretive synthesis (CIS) methods to review qualitative studies on patient experiences of Long Covid illness, recovery, and care. CIS methods prioritise sampling of rich and diverse data and are preferable in cases where the goal of the review is to generate theoretical insights and contributions beyond the findings of the original studies [23-25]. This requires critical, iterative, and reflexive approaches to developing a research question and undertaking sampling and analysis, rather than the systematic strategies utilised in many other review methodologies [24, 26]. We outline the steps of our methods below, which build upon an existing adapted approach to CIS methods [27].

Search strategy

We employed a combination of search strategies to identify literature for potential inclusion. First, we conducted searches of PubMed and Web of Science databases in September 2023 using analogous search queries (Supplemental Table 1). Additional publications were then identified through manual searches and citation chaining. As CIS methods do not require a systematically generated sample of literature, manual searching helped identify additional relevant studies to be considered for final inclusion in the analysis (Figure 1) [27, 28].

Inclusion

We developed four phases of sampling, adapted from an existing CIS approach [27]. These are presented in a simplified form in Figure 1; however, in practice these phases were not discrete or linear steps, but instead unfolded during the review process through reflexive, overlapping, and iterative practices of testing and analytical refinement. Eligibility for inclusion was kept intentionally broad in Phase I and was narrowed as we refined our

For peer review only

analytical focus, with additional criteria developed to support a higher overall interpretive value across the final sample [23]. Quality was appraised with a goal of maximising conceptual inclusion [23] using previously developed appraisal prompts [27] (Supplemental Table 2). Final inclusion was determined according to principles of conceptual saturation [23, 26], with studies included on the basis of contribution to theory development. Studies that did not produce any new insights from other included studies were excluded in Phase IV.

Our final sample included 16 studies and 879 participants with Long Covid or similar experiences (not accounting for potential duplication between studies), with most participants living in the UK or USA (Table 1). Primary study data was generated from these studies between July 2020 and May 2022. Most studies generated qualitative data through semi-structured interviews or focus groups (of samples ranging from 13 to 114 participants recruited via a combination of surveys, cohorts, social media, and clinical or community networks) [29-42] with two studies additionally generating visual data through participant illustration [38] and artist interpretation [41] methods. Other studies generated qualitative data via electronic health records (N=200) [38] and open-ended responses from a larger observational online survey (N=169) [43]. Studies used interpretive or descriptive thematic analysis methods, with half of included studies developing sociologically informed theoretical frameworks [30, 32, 35, 36, 38-41].

Table 1: Included studies

First author (year)	Country	Sample description (N)	Definition of Long Covid to determine study eligibility	Data collection period	Data description
Aghaei (2022) [29]	USA	Female long haulers (15)	Infected with Covid-19 and having experienced at least one Covid-19 symptom lasting four weeks or longer after a Covid-19 diagnosis	April to mid-June 2021	Interviews
Bergmans (2023) [30]	USA	Black patients with Long Covid (15)	Physical or mental health symptoms that lingered over one month after an acute Covid-19 infection	May to September 2021[1]	Interviews
Burton (2022) [31]	UK	People who self-reported Long Covid (21)	A positive swab test/antibody test or one or more commonly reported Covid-19 symptoms at illness onset (persistent cough, loss or change in taste/smell, high temperature); and experiencing one or more broader symptoms ≥3 weeks following the onset of their first symptoms	November 2020 to September 2021	Interviews
Callan (2022) [32]	UK	People with self-defined Long Covid and people from an online support group dedicated to Long Covid's	Not specified	October to November 2020	Focus groups

For peer review only - http://bmjopen.bmj.com/site/about/guidelines.xhtml

		neurocognitive effects (50)			
Chasco (2022) [33]	USA	Patients of a Midwestern academic hospital's post-Covid-19 clinic (15)	Persistent health concerns more than 3 months after SARS-CoV-2 infection	July to October 2021	Interviews
Cooper (2023) [34]	UK	People from a West London Long Covid clinical assessment centre (13)	Documented history of SARS-CoV-2 infection and reported persistent or new symptoms (> 12 weeks) following Covid-19	May to July 2021	Interviews
Fang (2023) [35]	UK	People self-identified as experiencing Long Covid symptoms (80)	Reported Covid-19-related symptoms for over 4 weeks, particularly focused on those who indicated having persistent symptoms for over 8 weeks	November 2021 to March 2022	Interviews
Maclean (2023) [36]	UK, USA, Netherlands, Canada, Australia	People with (Long) Covid (72)	Not specified	November 2020 to March 2022	Interviews
Moretti (2022) [37]	Italy	Women with Long Covid (17)	Experiencing Long Covid symptoms for at least three months following confirmed Covid-19 infection; having perceived a significant impact of Long Covid symptoms on quality of life; having consulted multiple healthcare professionals in order to receive a diagnosis	November to December 2021	Interviews
O'Brien (2023) [38]	Canada, USA, Ireland, UK	Adults who self-identified as living with Long Covid (40)	Signs and symptoms that develop during or following an infection consistent with Covid-19 that continue for 12 weeks or more and are not explained by an alternative diagnosis	December 2021 to May 2022	Interviews and participant visual illustrations
O'Hare (2022) [44]	USA	Veterans with Long Covid (200)	Documentation of a positive result on a polymerase chain reaction (PCR) test for SARS-CoV-2 and an ICD-10 diagnostic code for Long Covid	February to May 2022	Electronic health records
Ruthforth (2021) [39]	UK	People with Long Covid (114)	Not specified	May to October 2020	Interviews and focus groups
Russell (2022) [40]	USA	Self-identified long-haulers from online Covid-19 communities (20)	Long-term symptoms and effects from Covid-19 with one or more consultations with a healthcare professional about Covid-19 symptoms	March to April 2021	Interviews
Schmachtenberg (2023) [41]	Germany	Long Covid patients (25)	Symptoms that develop during or after an infection consistent with Covid-19, continue for more than 4 weeks, and are not explained by an alternative diagnosis	January to May 2022	Interviews and graphical artist interpretations
Taylor (2021) [42]	UK	Doctors with Long Covid (13)	Not specified	July to August 2020	Interviews

For peer review only - http://bmjopen.bmj.com/site/about/guidelines.xhtml

Wurz (2022) [43]	International (92% from UK, USA, or Canada)	Adults living with Long Covid (169)	Currently experiencing long-term symptoms due to Covid-19 (at least 4 weeks since the acute illness or positive Covid-19 test, with symptoms not predating the acute illness); and having tested positive for Covid-19, or with probable infection (based on an illness mimicking the acute phase of Covid-19, having close contact with a confirmed case, or being linked with an outbreak), in line with the clinical case definition for post-Covid-19 condition	February to April 2021	Open-ended responses in online survey
[1] This is the period of participant recruitment, as the period of data collection was not specified.					

Data extraction and analysis

Emerging themes were identified and manually coded in Phase II of sampling (Figure 1). These early themes guided the development of additional eligibility criteria, which were used to refine the final sample based on contribution to the conceptual goal of the synthesis. Through an iterative process of sampling and preliminary analysis, synthetic constructs were developed to organise the conceptual argument. Synthetic constructs allow for both underlying evidence and second order analyses in included studies to be assembled, critically analysed, and transformed, with a goal of developing new conceptual arguments and analytical insights [23]. We thus approached data analysis of included studies at two orders: analysis of the primary data presented in the articles and analysis of the interpretations of primary data developed by the authors of the studies [23, 27].

Following Dixon-Woods et al. [23], our analyses of included studies develop a synthesising argument that is grounded in the data yet extends upon the findings of included studies to develop new insights and make sense of the research in a novel and explanatory way. This synthesising argument assembles both our synthetic constructs and second order interpretations of data presented by included studies. This is an established approach to CIS methods [23, 27] but is especially valuable for our analysis of Long Covid. A key concern has been how Long Covid is studied and understood by academics, clinicians, patients, and activists [4, 5, 7, 21]. Our critical interpretation of study authors' interpretations of Long Covid data is therefore not only an appropriate approach to CIS analysis; it also constitutes a crucial line of inquiry for investigating how Long Covid has become known, diagnosed, experienced, researched, and cared for as an emerging, uncertain, and contested health condition.

Patient and Public Involvement

There was no patient or public involvement in this study.

For peer review only - http://bmjopen.bmj.com/site/about/guidelines.xhtml

Findings

Our synthesis investigates lived experiences of Long Covid. We organise our analysis into three thematic constructions of Long Covid: the illness, the patient, and recovery. Table 2 presents illustrative participant extracts from included studies, organised by theme and subtheme.

Table 2: Illustrative participant extracts from studies

Theme	Subtheme	Example Quote
The illness	Complex symptom presentation	"I had an odd rash for quite a while it kept coming and going ... very itchy cough ... very mild asthma [...] I just kept putting it down to grief until a couple of months in a friend said, 'Look do you think this could be Covid?'" [39]<br><br>"The biggest challenge is not knowing when [the episodes are] going to happen, how long they're going to last or how much I can get away with before causing it, if I am truly the cause of one of these episodes." [38]
	Symptoms as inherently strange	"It's been rather mysterious [...] and it doesn't feel like anything else, it's bizarre." [42]<br><br>"The fatigue is not exactly tiredness as in sleepiness. It is fatigue as in how a healthy person would feel the day after they ran a marathon." [43].
	Hiding implausible symptoms	"I'd often get a heart rate of 130, just randomly watching TV. And avoided telling my GP about that. [...] I didn't want to admit it because I think I was worried about it being dismissed as anxiety." [42]<br><br>"I thought had gotten better, so you know, the phantom smells, the tinnitus, I'd had some strange nerve pain, like a poker in my ear, really bizarre, but it was very, very painful. Strange kind of scalp sensations, as well, but all, all such vague and strange things, I didn't even mention them to, to my GP, because I thought she might think I was completely mad." [36]

For peer review only - http://bmjopen.bmj.com/site/about/guidelines.xhtml

	Fuzzy symptom categories and ambiguous causation chains	"I have constant headaches, that is true, but I don't know if that is because of COVID or just because I'm stressed, it is psychological." [34]<br><br>"She also relates a history of asthma and chronic cough. Difficult at this time to separate out post COVID recovery from potential underlying asthma." [44]
The patient	Thresholds of symptom severity for patienthood	"I feel like it probably doesn't apply to me, because I feel like my symptoms, or whatever I have left, is not as severe as what other people are going through." [34]<br><br>"I don't want to put any pressure on [the] health service. [It] is quite awful to come and approach a GP and say, 'I'm really tired.'" [36]
	Disrupted daily life as evidence of patienthood	"I used to go out dancing twice a week […] and you think, well, I couldn't even get up, let alone go dancing twice a week... You reach a point where you don't actually remember being well." [35]<br><br>"I had COVID and five months later, I'm still not better. […] I can't look after my children for long periods of time, or in the initial stages at all, and that created huge problems." [42]
	Health professionals as authenticators of patienthood	"I have come across a few doctors where I've felt they've made me feel a bit stupid when I'm worrying about certain things ... So yes, there's that worrying that people just think you're making it up." [31]<br><br>"[The doctor] listened to absolutely everything I had to say, every weird symptom that I thought was unrelated. You know, he listened to everything and then would tell me, 'Nope, that's all part of it.'" [36]
	Managing care through self-experimentation	"I have gone all out on the quackery, which I never thought I would because I've never been that person. I've had acupuncture a few times. I am under a dietician and I'm taking all sorts of weird and wonderful supplements." [42]

For peer review only - http://bmjopen.bmj.com/site/about/guidelines.xhtml

		"I even let my daughter and I let my friend, my mom, wear my Apple Watch for a day. Like, 'Let me see if [the high heart rate notification] goes off for you.' If it's not going off for you, then why is it going off for me?" [30]
Recovery	Illness and recovery as temporary	"You don't feel good, but you have to get to work, and then you have kids and family. […] You are expected to do [it] all." [29]<br><br>"I also had two colleagues who complained about me to the staff council, along the lines of 'I always have to cover for her because she is sick.'" [41]
	Non-linear recovery trajectories	"I liken it to a rollercoaster ride and you're like up and down... there are good days and bad days.... my symptoms are still there but it kind of goes down." [38]<br><br>"You feel like shit for a few days [with Covid-19], you start to feel a little bit better, then you feel like shit again […] it's about a whole new experience of illness. […] You come to doubt your knowledge of illness and recovery." [36]
	Illness/wellness binaries	"In March, we thought COVID was something that would either kill me in three weeks or I'd get over it ... It was May when I started having long-term symptoms. […] Am I the only one going through this or are there others?" [40]<br><br>"It's like when somebody has a stroke, and they get partially paralysed. […] They might regain partial control, like learn to walk again, but their arm might not ever be working again." [40]
	Indeterminacy of acute/Long Covid recovery	"The Long Covid name to me, feels like you've still got COVID; it's like a long version of it. […] I always say post COVID, personally, just because usually when talking about things like post-op, you would say, not long-op." [34]

For peer review only

		"It's a bit wishy-washy, to me anyway, but also, I think everyone is feeling that they've got slightly different as well. […] It's a bit of a broad term, isn't it?" [34]
	Restorative versus chronic models of illness	"I would rather [the term Long Covid] than say, chronic COVID, that would be a lot more worrying." [34]<br><br>"I consider myself disabled 24/7. […] I'm always at this minimal state, […] it's not like I go from 'oh today I can do all these things.' No, there's no day like that." [38]

For peer review only

The illness

The first question that studies on Long Covid have addressed is: 'what is Long Covid'? All but two of the included studies were published in 2022 or 2023, yet the complexity of this question remains a consistent concern. Studies defined Long Covid using clinical guidelines and criteria (such as those of the World Health Organization and the UK National Institute for Health and Care Excellence) [31, 38, 41, 42], as well as on the basis of self-identification, noting the role of patient advocacy in the naming of Long Covid [32, 34-36, 39, 40, 43, 44]. The term Long Covid was generally used, though studies also acknowledged other labels such as "post-Covid syndrome," "Post-Acute Sequelae of Covid-19 (PASC)," and "long haulers" [29, 31-34, 37-44]. One study made explicit distinction in the use of terms, using "long COVID-19" to describe "the lived patient experience," and "post-COVID-19 syndrome" to describe "the medically diagnosed condition" [32].

These working definitions of Long Covid informed participant inclusion criteria, which varied across the studies (Table 1). Most studies identified a minimum number of weeks that participants must have experienced persistent or new symptoms after acute illness (usually 4 or 12 weeks) [29-31, 33-35, 37, 38, 41, 43], with a subset stating preference for confirmed SARS-CoV-2 infection, such as through a positive test or a diagnosis from a clinician [31, 34, 43]. One study required a formal Long Covid diagnosis from a clinician [44]. A handful of studies determined eligibility through self-identification or did not define Long Covid in their reported eligibility criteria [32, 36, 39, 42].

All studies described Long Covid as a complex condition consisting of many and varied symptoms. While none of the included studies identified specific symptoms or illness effects as requisites for (or not included within) Long Covid diagnoses, the symptomology and aetiology of Long Covid were primary concerns. All studies we reviewed described specific symptoms experienced by participants, with some studies additionally tracking how common particular symptoms were across the study sample [30, 31] or how long symptoms had persisted [30, 31, 33, 36, 38, 40, 43]. Symptoms tended to be described in generally

For peer review only - http://bmjopen.bmj.com/site/about/guidelines.xhtml

recognisable terms (such as "brain fog," "fatigue," "headaches," "shortness of breath"), with a focus on how symptoms were managed and impacted on participants' daily lives.

Yet in several studies, participants also emphasised the "strangeness" of their symptoms, suggesting that commonly used terms inadequately described their embodied experiences [36, 39, 40, 42, 43]. Recognising these "strange" symptoms in others made Long Covid legible as a heterogeneous illness with which people of various experiences could collectively identify. When these diverse presentations were not widely recognised or accepted (especially early in the pandemic), some participants avoided disclosing symptoms they considered to be "implausible" [36, 42]. Other participants made sense of complex Long Covid effects through comparisons to health conditions with better understood or more widely accepted biological mechanisms, such as stroke or traumatic brain injury [32, 34, 40, 42].

Several studies emphasised that symptoms should not have predated Covid-19 infection or be explained by "alternative diagnoses" [38, 41, 43, 44]. Yet in practice, the presentation of Long Covid symptoms was often fuzzy and revealed slippages between descriptions of the symptoms of Long Covid and its extended effects. This was particularly common with psychological and mental health symptoms, with some studies describing symptoms such as anxiety and depression as direct manifestations of Long Covid, and others framing mental health impacts as part of a more complex chain of illness effects, for instance as a consequence of fatigue [29-31, 33, 34, 37, 43]. These ambiguous causation chains also intersected with the broader pandemic context [34]. In cases where participants had a pre-existing disability or health condition, experiences of Long Covid were sometimes described as an "exacerbation" of these conditions, further complicating the actualisation of Long Covid as a distinct and traceable illness [30, 34, 38, 44].

The patient

Because of the complexity and diversity of Long Covid symptoms, another challenge of characterising Long Covid was determining who counts as a Long Covid 'patient' (and in what situations). A priori study eligibility criteria inevitably stabilise the constitution of the Long Covid patient to some extent, yet our analysis of studies nonetheless indicates heterogeneity. For example, some participants referenced an imagined threshold of severity at which a person becomes 'sick enough' to become a 'Long Covid patient' (as defined by themselves or by healthcare professionals) [34, 40]. Overall, however, the 'patient' of Long Covid was constituted in studies according to two qualifiers: symptoms that brought about *disruption* in daily life; and people in need of *care* as a consequence of their symptoms.

Studies generally evidenced "disruption" in daily life in relation to the capacity of participants to maintain an active social life [29, 31, 35, 37, 38, 41, 43], return to work [29, 31-33, 35, 37, 40-43], engage in physical activity and exercise [29-31, 33-35, 41, 43], or carry out domestic tasks [29, 31, 33, 35, 36, 38, 41, 43]. For example, "fatigue" was described in several studies in terms of participants having insufficient stamina to play the sports they enjoyed prior to Covid [30, 31, 35, 41, 43]. The boundaries of these illness effects were fuzzy and complex, with Long Covid's disruptiveness intersecting with multiple other social and

For peer review only - http://bmjopen.bmj.com/site/about/guidelines.xhtml

environmental factors, such as when patienthood precluded the assumed caring responsibilities of motherhood [29, 30].

Many studies framed experiences of seeking care for Long Covid in relation with other experiences of “chronic illness,” “hidden disability,” and “contested illness,” including through comparisons to ME/CFS, HIV-related illnesses, or fibromyalgia [32, 33, 35-40, 42-44]. Management of Long Covid via formal health services was described as “fragmented,” insufficiently resourced, “poorly coordinated,” and lacking relevant knowledge or treatment options [31, 36, 37, 39, 40, 42, 44]. Participants expressed frustration at being expected to develop their own treatment plans and insufficient or ambiguous diagnostic and monitoring technologies for “making sense” of their Long Covid illness experience [36-39, 42]. Participants also reported difficulty in understanding processes for securing referrals to appropriate specialists [31, 36, 39], especially if neurological issues made it harder for them to advocate for themselves and explain their experiences [32]. Other barriers to accessing services included requiring formal diagnoses or proof of positive Covid-19 tests [31, 36, 40] or changes in eligibility criteria [31]. Some participants also described feelings of “guilt” for accessing an under-resourced health system, especially when people were dying from Covid-19 [36, 42].

Another care-related concern described in studies was in relation to the diagnostic category of Long Covid. Studies described experiences of medical “gaslighting” (especially earlier in the pandemic), where participants had to try to “prove” the reality of their illness and felt that their experiences were often “minimised” or “dismissed as anxiety” [30-32, 35-37, 39, 40, 42]. Participants emphasised the importance of having “weird” symptoms “validated” by medical professional as part of Long Covid and described this validation as a form of care in itself [32, 34, 36, 39, 42]. Participants also reported mixed experiences of care and support from family and friends, noting similar challenges to that of formal care seeking such as insufficient knowledge and resources, or “disbelief” [29, 30, 33, 35, 37, 39]. For example, some participants found initial support from friends and family became strained over time [31], especially when symptoms were “invisible” or “unpredictable” [38].

Across studies, care was actualised via “self-management,” self-investigation, and “hypothesising” about Long Covid illness. For instance, some studies described management of Long Covid through processes of embodied experimentation, such as through trying different diets, pharmacological interventions, vitamins and supplements, or alternative therapies [30, 32, 34, 37, 42, 43]. Many participants described managing their illness as a series of “trade-offs” or “compromises” where they worked out what they had the energy to do each day [29, 30, 32, 34, 38, 39, 43]. These experiments required participants to monitor their bodies to determine what was working or what could be triggering particular illness effects. Several participants also engaged in collective “sense-making” and knowledge generation via online Long Covid communities [30, 31, 36, 39, 40]. While these communities provided practical support and validation, participants in one study highlighted the potential dangers in these cultures of knowledge sharing, which could include “unverified medical advice” [40].

For peer review only - http://bmjopen.bmj.com/site/about/guidelines.xhtml

Recovery

Studies presented the experience of Long Covid recovery as marked by “uncertainty” [30-32, 36-38, 40, 44]. While some participants described recovery as an incremental process towards becoming well again [32, 43], far more studies included participants who described recovery in terms of “episodic illness” or “disability” [32, 38, 39, 43] that was “unpredictable” in nature and marked by “relapses” or periods of worsened symptoms [31-34, 36, 38-40, 43, 44]. This episodic experience of Long Covid disrupted narratives of recovery as a “linear pathway” from ‘sick’ to ‘better’ [36, 38-40, 42, 44]. Participants who had experienced Long Covid earlier in the pandemic found this particularly troubling, as their lived experiences of Long Covid differed greatly from the public understanding of Covid-19 illness in which the danger was perceived to lie in whether or not the acute illness caused death [40, 42]. Some participants also described an initial period of “recovery” following acute illness or periods of “partial recovery” from Long Covid, which troubled this trajectory further [34, 39].

The chronicity of recovery raised ontological questions about the relationship between acute illness and Long Covid. Long Covid was characterised by some participants as existing on a “continuum” from the time of acute illness, while others saw it as “fluctuating,” and organised as “clusters” or “coming in waves” [34, 38, 39]. One study described conflicting participant perspectives as to whether Long Covid existed at all as a discrete illness or condition, with some participants describing their experiences as simply a period of recovery from Covid-19, as one might expect following any flu-like illness [34]. Others described Long Covid as an especially significant experience of recovery that might not ever result in a full return to their previous health [40]. The distinction between recovery from acute illness and Long Covid was complicated further when relapses resulted in hospitalisation, which could produce additional illness events from which to recover [44].

The terminology of Long Covid also related to how recovery was characterised. For some participants, labels like “Long Covid” validated their experiences as distinct from other illnesses (such as acute Covid-19 or ME/CFS) and as a recognisable illness shared with other people [33, 34, 36, 37]. However, one study described some participants feeling as though the label implied that their experiences were part of the *same* illness as acute Covid-19 [34]. Other language rendered the chronicity and ontology of Long Covid similarly complex. For example, some participants identified as being “disabled” or having a “chronic” illness or health condition [33, 35, 38, 40, 43]. These terms designated Long Covid as a stable and ongoing condition, rather than something to be recovered from (or a process of recovery in and of itself), which was a shift in framing that some participants found distressing [34, 35].

These experiences all intersected with expectations of what recovery ‘should’ look like. Participants described social and professional pressures to “recover” and return to “normal” [29, 30, 33, 41]. Participants’ “guilt” about being unable to perform their previous social roles was compounded when they additionally required care from their family or partner [29, 33, 41]. Some participants internalised these expectations and questioned their own sense of what it means to recover from illness [36, 39, 40]. One study summed up the

For peer review only - http://bmjopen.bmj.com/site/about/guidelines.xhtml

conflict between expectations of recovery and the lived experience of Long Covid illness as a tension between the roles of Long Covid patient and “Covid-19 survivor” [29].

Discussion

Our synthesis generates three intersecting analytical themes relating to the epistemic construction of Long Covid: the illness, the patient, and recovery. While these themes were present across all studies, we emphasise that the constitution of Long Covid across these themes produces different implications for the research, management, and lived experience of Long Covid. Our discussion highlights how this constitutive work was done in and by studies, and its effects. It furthermore accentuates that a flexible yet situated approach to defining Long Covid enables the development of more critical research and health approaches that acknowledge a diversity of Long Covid experiences and care needs.

Reflections on study design

The design and methods of qualitative study constitute the object of Long Covid and thus how Long Covid comes to be understood in specific ways. Studies variably framed Long Covid as a diagnostic category, a lived experience of illness or recovery, a social identity, a medical object of research or care, a policy concern, and more. Studies also defined Long Covid in relation to global health guidelines and/or accounts emerging through patient advocacy, which diversely characterised Long Covid; for example, Table 3 presents descriptors that were *applied to* and *demarcated from* definitions and accounts of Long Covid across the studies. Inconsistencies between these characterisations of Long Covid (as well as across lived experiences and clinical interpretations described within study data) were generally navigated by framing Long Covid as an “uncertain,” “contested,” or “emerging” illness [32, 34-37, 40, 42, 43].

Table 3: Descriptors used in Long Covid in studies

- Acute Covid-19 illness
- Recovery following acute Covid-19 illness
- Post-Covid Syndrome or a similar formal diagnosis
- Extended effects of acute Covid-19 illness, hospitalisation, and/or rehabilitation including due to being unable to exercise or being on a ventilator
- Covid-19 symptoms persisting for a specified minimum length of time following onset of Covid-19
- Symptoms with understood and verifiable underlying biological mechanisms
- A discrete list of recognised and validated Long Covid symptoms
- Symptoms of pre-existing illness or disability that are exacerbated following Covid-19 illness
- Mental illness or psychological distress
- Chronic illness or disability

For peer review only - http://bmjopen.bmj.com/site/about/guidelines.xhtml

- A syndrome or collective label for many different illness experiences
- Symptoms that cannot be explained by other diagnoses
- ME/CFS

The constitution of Long Covid within participant eligibility criteria similarly varied across study methods. Most studies recruited participants through Long Covid support groups or by publicly advertising the study, though some [33-35, 44] sampled from clinical settings or existing cohorts, which enabled researchers to identify eligible participants from a broader repository of health data. All recruitment strategies required participants, researchers, or clinicians to identify Long Covid patients, which meant that inclusion was shaped by evolving understandings and public awareness regarding the constitution of Long Covid at the time of recruitment. Future qualitative studies need to be more open in their iterative sampling and exploration so as not to close-off or narrow Long Covid as a site which is emergent in its experience, including through how it is defined and understood beyond clinical and biomedical understandings.

Studies in our synthesis also orientated largely in relation to 'realist' and/or 'critical realist' approaches using descriptive and/or interpretive thematic analyses of semi-structured interview data. A minority of synthesised studies adopted 'critical' and/or 'social constructionist' approaches [32, 35-37, 39-41], focused for instance, on how narratives 'enact' or 'perform' constitutions of Long Covid [32, 35, 36, 39, 40]. Our broader review of qualitative Long Covid research found that many studies have been developed as an adjunct to surveys, cohorts and/or clinical studies, rather than designed as stand-alone qualitative studies in their own right. There is greater scope for critical sociological qualitative analyses in this field, especially those which treat narrative as a process of enactment for study rather than simply as a resource of lived experience description [45, 46]. The constitution of Long Covid as an emergent, situated, and negotiated lived embodiment emphasises the importance of qualitative designs which approach illness, patienthood, and recovery as matters of 'construction' and 'enactment.'

From Long Covid symptoms to Long Covid patients

Our synthesis highlights a significant emphasis in studies on identifying and measuring Long Covid 'symptoms' in relation to physical manifestations of illness, which are largely clinically defined. Studies we reviewed did not identify *specific* symptoms as proving or precluding a Long Covid diagnosis or experience. Instead, Long Covid emerges in our synthesis as an open category of coalescing symptoms which find meaning in their relations with acute Covid-19 infection, time, and an embodied sense of disruption to a 'normal.'

Studies accentuated the temporality of Long Covid as an open category that included symptoms felt to be persistent or recurring following acute Covid-19 infection, symptoms felt to be new and different, as well as symptoms which could come and go over time and with varying degrees of severity. Studies used different minimum timeframes of recurring or ongoing symptoms as a condition of Long Covid, yet the 'long' nature of Long Covid was

For peer review only - http://bmjopen.bmj.com/site/about/guidelines.xhtml

consistently identified as a defining feature of the illness experience and without a clear end point.

The constitution of Long Covid as a disruption to the 'normal' was similarly oriented towards symptoms across studies. Studies noted, and some deployed in their inclusion criteria [38, 41, 43], Long Covid symptoms as unexplainable by alternative diagnoses. However, we found that otherwise familiar symptoms, including from existing medical or health conditions [30, 34, 38, 44], were made 'strange' in studies through complex symptom clusters, heightened illness effects, and unpredictable recovery trajectories that disrupted 'normal' life [36, 39, 40, 42, 43].

Furthermore, ambiguities surrounding emergent and speculative symptom causation show that experiences of Long Covid are not merely 'physical' but 'social'; we found the boundaries between Long Covid's causation, its disruptive effects on life, and the broader complex social worlds of patients (which include experiences of global pandemic) were often fuzzy and variably defined [29-31, 33, 34, 37, 38, 43, 44]. This fuzziness made responding to and caring for Long Covid (in formal and informal settings) difficult and could result in participants feeling they had been denied Long Covid patienthood [30-32, 35-40, 42].

These inconsistencies and complexities in Long Covid criteria have been subject to critique [4, 5]. Yet the interpretation we advance through our synthesis suggests that many of the challenges related to more open and changeable constitutions of Long Covid emerge as a result of insufficiently situating this constitutive work. For example, our synthesis attests to the utility of Long Covid as a diagnostic label to validate and help make sense of illness experience in people's lives and social worlds, even in the absence of clinical care provision [30-32, 34, 36, 39, 40, 42]. There is a need for researchers and care providers to embrace broader and more open understandings of Long Covid 'symptoms' and 'diagnosis' that reflect the diversity of experiences examined across qualitative studies—not to capture more people within a singular definition of the 'Long Covid patient' with standardised 'care' needs, but to acknowledge the complex reality of Long Covid experiences.

The object of Long Covid recovery

Like Long Covid patienthood, recovery is multiply interpreted across the studies we reviewed. Our synthesis suggests that non-linear trajectories of Long Covid recovery, where illness does not become progressively better over time but is instead marked by unexpected or relapsing symptoms, is experienced as a disruption of recovery itself [38-40, 42, 44]. This produces new health uncertainties when expectations of what Long Covid recovery 'should' look like fail to materialise [29, 30, 33, 36, 40-42]. Across studies, these effects were most noticeable for participants who had experienced Long Covid earlier in the pandemic when there was less knowledge about post-Covid-19 illness and recovery [31, 32, 36, 39, 40, 42].

The non-linearity of Long Covid experiences also challenged notions of 'full' recovery or 'partial' recovery. Some participants experienced Covid-19 recovery as a separate process to Long Covid (recovery) [34, 39] and others resisted assumptions of recovery entirely, describing themselves as disabled [33, 35, 38, 40, 43]. Here our synthesis resonates with

For peer review only - http://bmjopen.bmj.com/site/about/guidelines.xhtml

existing research on experiences of chronic illness, which argues that so-called 'restoration' of bodily capacities following illness and disability is not always realistic or possible [47, 48]. Furthermore, while diagnostic labels can give patients "permission to be ill" [49, 50], our findings suggest that this social permission has been predicated on an assumption that illness is temporary and that patients will soon resume 'normal' professional and social activities [29, 30, 33, 41]. Such conditional freedom from social obligations also mirrors broader experiences of chronic illness and disability [47, 51, 52].

As observed in other social and health research contexts [53-55], diverging understandings of what constitutes illness and recovery produce challenges for designing research and health interventions. Our analysis identifies multiple health 'problems' from which participants were described as recovering, including SARS-CoV-2 viral infection, Covid-19 illness (and in some cases hospitalisation), Long Covid, or something less precise and entangled with broader social factors and the pandemic context. Processes of recovery were also diversely characterised. While a 'return to normal' was both possible and desirable for some, recovery was also characterised as an unpredictable process requiring ongoing care, a process of working towards a new version of normal, or was rejected entirely.

These insights highlight the importance of interrogating the problem and goal of care and recovery when discussing Long Covid. Notions of care, patienthood, recovery, and being 'recovered' held different meanings for participants across studies, which had implications for how the experience of Long Covid became understood—for participants, their clinicians, people in their social and professional lives, and through the studies themselves. While one person's definition of being recovered from (Long) Covid might require the complete absence of symptoms or illness effects, others might feel recovered when they feel well enough to work or no longer require frequent medical interventions. Our synthesis emphasises that classifications of Long Covid as an illness must be contextualised in relation with specific interpretations of patienthood and recovery if they are to be meaningfully studied and targeted in health interventions.

Conclusion

Through this synthesis, we accentuate Long Covid as a site of multiple qualitative interpretation, giving rise to coexisting versions of illness, patienthood, and recovery. Crucially, we find that Long Covid develops meaning through its social relations of varied and diverse lived experience. We therefore caution against attempts to align or stabilise constitutions of Long Covid illness, patienthood, and recovery in a singular trajectory or understanding. Accounts of patients' experiences illuminate how illness, patienthood, and recovery are sites of continual negotiation and thus also defining qualitative features of what Long Covid is. Efforts to diagnose and intervene must be as adaptive as the varieties of Long Covid lived experience.

For peer review only

Acknowledgements

We acknowledge funding support from the UK National Institute for Health and Care Research (grant NIHR135315). The Centre for Social Research in Health at the University of New South Wales (UNSW) is supported by a grant from the Australian Government Department of Health. We are grateful for support from the UNSW SHARP (Professor Tim Rhodes) and Scientia Fellowship (Associate Professor Kari Lancaster) schemes. Kari Lancaster is supported by an Australian Research Council DECRA Fellowship (DE230100642).

Author contributions

MH led the conception and design of the review, data collection, and analysis. TR and KL contributed to research design and analysis. MH drafted the manuscript, with all authors contributing to write-up and revision.

Funding

We acknowledge funding support from the UK National Institute for Health and Care Research (grant NIHR135315). We are grateful for additional support from the UNSW SHARP (Professor Tim Rhodes) and Scientia Fellowship (Associate Professor Kari Lancaster) schemes.

Competing interests

None declared.

Data availability statement

Not applicable.

Ethics approval

Not required—review. Ethics approval linked to grant NIHR135315 was granted via the London School of Hygiene and Tropical Medicine Observational Research Ethics Committee (Ref 28635).

Figure Legend

Figure 1: Flow diagram of phases of sampling for qualitative interpretative synthesis, adapted from existing CIS approach [27].

For peer review only

References

1. Cabrera Martimbianco AL, Pacheco RL, Bagattini ÂM, Riera R. Frequency, signs and symptoms, and criteria adopted for long COVID-19: A systematic review. International Journal of Clinical Practice. 2021;75(10):e14357. DOI: 10.1111/ijcp.14357
2. Albright C, Limoges J, Rempel GR. Living with pulmonary sequelae of COVID-19 and the implications for clinical nursing practice: A qualitative systematised review. Journal of Clinical Nursing. 2023;0(0):1-11. DOI: 10.1111/jocn.16664
3. Macpherson K, Cooper K, Harbour J, Mahal D, Miller C, Nairn M. Experiences of living with long COVID and of accessing healthcare services: a qualitative systematic review. BMJ Open. 2022;12(1):e050979. DOI: 10.1136/bmjopen-2021-050979
4. Roth PH, Gadebusch-Bondio M. The contested meaning of “long COVID” – Patients, doctors, and the politics of subjective evidence. Social Science & Medicine. 2022;292:114619. DOI: 10.1016/j.socscimed.2021.114619
5. Barker KK, Whooley O, Madden EF, Ahrend EE, Greene RN. The long tail of COVID and the tale of long COVID: Diagnostic construction and the management of ignorance. Sociology of Health & Illness. 2022. DOI: 10.1111/1467-9566.13599
6. Greenhalgh T, Knight M, A’Court C, Buxton M, Husain L. Management of post-acute covid-19 in primary care. BMJ. 2020;370:m3026. DOI: 10.1136/bmj.m3026
7. Hunt J. Making space for disability studies within a structurally competent medical curriculum: reflections on long Covid. Medical Humanities. 2023;49(1):105-16. DOI: 10.1136/medhum-2022-012415
8. Almas T, Malik J, Alsubai AK, Jawad Zaidi SM, Iqbal R, Khan K, et al. Post-acute COVID-19 syndrome and its prolonged effects: An updated systematic review. Annals of Medicine and Surgery. 2022;80:103995. DOI: 10.1016/j.amsu.2022.103995
9. Iqbal FM, Lam K, Sounderajah V, Clarke JM, Ashrafian H, Darzi A. Characteristics and predictors of acute and chronic post-COVID syndrome: A systematic review and meta-analysis. EClinicalMedicine. 2021;36:100899. DOI: 10.1016/j.eclinm.2021.100899
10. Nguyen NN, Hoang VT, Dao TL, Dudouet P, Eldin C, Gautret P. Clinical patterns of somatic symptoms in patients suffering from post-acute long COVID: a systematic review. European Journal of Clinical Microbiology & Infectious Diseases. 2022;41(4):515-45. DOI: 10.1007/s10096-022-04417-4
11. Michelen M, Manoharan L, Elkheir N, Cheng V, Dagens A, Hastie C, et al. Characterising long COVID: a living systematic review. BMJ Global Health. 2021;6(9):e005427. DOI: 10.1136/bmjgh-2021-005427
12. Akbarialiabad H, Taghrir MH, Abdollahi A, Ghahramani N, Kumar M, Paydar S, et al. Long COVID, a comprehensive systematic scoping review. Infection. 2021;49(6):1163-86. DOI: 10.1007/s15010-021-01666-x
13. Kopanczyk R, Kumar N, Papadimos T. Post-Acute COVID-19 Syndrome for Anesthesiologists: A Narrative Review and a Pragmatic Approach to Clinical Care. Journal of Cardiothoracic and Vascular Anesthesia. 2022;36(8, Part A):2727-37. DOI: 10.1053/j.jvca.2021.09.051
14. van Kessel SAM, Olde Hartman TC, Lucassen PLBJ, van Jaarsveld CHM. Post-acute and long-COVID-19 symptoms in patients with mild diseases: a systematic review. Family Practice. 2021;39(1):159-67. DOI: 10.1093/fampra/cmab076

For peer review only

For peer review only - http://bmjopen.bmj.com/site/about/guidelines.xhtml

15. Wong TL, Weitzer DJ. Long COVID and Myalgic Encephalomyelitis/Chronic Fatigue Syndrome (ME/CFS)—A Systemic Review and Comparison of Clinical Presentation and Symptomatology. Medicina. 2021;57(5):418. DOI: 10.3390/medicina57050418
16. Fugazzaro S, Contri A, Esseroukh O, Kaleci S, Croci S, Massari M, et al. Rehabilitation Interventions for Post-Acute COVID-19 Syndrome: A Systematic Review. International Journal of Environmental Research and Public Health. 2022;19(9):5185. DOI: 10.3390/ijerph19095185
17. Rinn R, Gao L, Schoeneich S, Dahmen A, Anand Kumar V, Becker P, et al. Digital Interventions for Treating Post-COVID or Long-COVID Symptoms: Scoping Review. J Med Internet Res. 2023;25:e45711. DOI: 10.2196/45711
18. Yong SJ, Liu S. Proposed subtypes of post-COVID-19 syndrome (or long-COVID) and their respective potential therapies. Reviews in Medical Virology. 2022;32(4):e2315. DOI: 10.1002/rmv.2315
19. Hossain MM, Das J, Rahman F, Nesa F, Hossain P, Islam AMK, et al. Living with "long COVID": A systematic review and meta-synthesis of qualitative evidence. PLOS ONE. 2023;18(2):e0281884. DOI: 10.1371/journal.pone.0281884
20. Zheng X, Qian M, Ye X, Zhang M, Zhan C, Li H, et al. Implications for long COVID: A systematic review and meta-aggregation of experience of patients diagnosed with COVID-19. Journal of Clinical Nursing. 2022;0(0):1-18. DOI: 10.1111/jocn.16537
21. Callard F, Perego E. How and why patients made Long Covid. Social Science & Medicine. 2021;268:113426. DOI: 10.1016/j.socscimed.2020.113426
22. Mol A, Hardon A. What COVID-19 may teach us about interdisciplinarity. BMJ Global Health. 2020;5(12):e004375. DOI: 10.1136/bmjgh-2020-004375
23. Dixon-Woods M, Cavers D, Agarwal S, Annandale E, Arthur A, Harvey J, et al. Conducting a critical interpretive synthesis of the literature on access to healthcare by vulnerable groups. BMC Med Res Methodol. 2006;6(1):35. DOI: 10.1186/1471-2288-6-35
24. Booth A. Harnessing Energies, Resolving Tensions: Acknowledging a Dual Heritage for Qualitative Evidence Synthesis. Qual Health Res. 2019;29(1):18-31. DOI: 10.1177/1049732318808247
25. Booth A, Carroll C, Ilott I, Low LL, Cooper K. Desperately Seeking Dissonance:Identifying the Disconfirming Case in Qualitative Evidence Synthesis. Qual Health Res. 2013;23(1):126-41. DOI: 10.1177/1049732312466295
26. Barnett-Page E, Thomas J. Methods for the synthesis of qualitative research: a critical review. BMC Med Res Methodol. 2009;9(1):59. DOI: 10.1186/1471-2288-9-59
27. Harrison M, Rhodes T, Lancaster K. How do care environments shape healthcare? A synthesis of qualitative studies among healthcare workers during the COVID-19 pandemic. BMJ Open. 2022;12(9):e063867. DOI: 10.1136/bmjopen-2022-063867
28. Greenhalgh T, Peacock R. Effectiveness and efficiency of search methods in systematic reviews of complex evidence: audit of primary sources. BMJ. 2005;331(7524):1064-5. DOI: 10.1136/bmj.38636.593461.68
29. Aghaei A, Zhang R, Taylor S, Tam CC, Yang CH, Li X, et al. Social Life of Females with Persistent COVID-19 Symptoms: A Qualitative Study. Int J Environ Res Public Health. 2022;19(15). DOI: 10.3390/ijerph19159076
30. Bergmans RS, Chambers-Peeple K, Yu C, Xiao LLZ, Wegryn-Jones R, Martin A, et al. 'I'm still here, I'm alive and breathing': The experience of Black Americans with long COVID. Journal of Clinical Nursing. 2023. DOI: 10.1111/jocn.16733

For peer review only

31. Burton A, Aughterson H, Fancourt D, Philip KEJ. Factors shaping the mental health and well-being of people experiencing persistent COVID-19 symptoms or 'long COVID': qualitative study. Bjpsych Open. 2022;8(2). DOI: 10.1192/bjo.2022.38
32. Callan C, Ladds E, Husain L, Pattinson K, Greenhalgh T. 'I can't cope with multiple inputs': a qualitative study of the lived experience of 'brain fog' after COVID-19. Bmj Open. 2022;12(2). DOI: 10.1136/bmjopen-2021-056366
33. Chasco EE, Dukes K, Jones D, Comellas AP, Hoffman RM, Garg A. Brain Fog and Fatigue following COVID-19 Infection: An Exploratory Study of Patient Experiences of Long COVID. International Journal of Environmental Research and Public Health. 2022;19(23). DOI: 10.3390/ijerph192315499
34. Cooper E, Lound A, Atchison CJ, Whitaker M, Eccles C, Cooke GS, et al. Awareness and perceptions of Long COVID among people in the REACT programme: Early insights from a pilot interview study. Plos One. 2023;18(1). DOI: 10.1371/journal.pone.0280943
35. Fang C, Baz SA, Sheard L, Carpentieri JD. 'I am just a shadow of who I used to be'—Exploring existential loss of identity among people living with chronic conditions of Long COVID. Sociol Health Illn. 2023. DOI: 10.1111/1467-9566.13690
36. Maclean A, Hunt K, Brown A, Evered JA, Dowrick A, Fokkens A, et al. Negotiation of collective and individual candidacy for long Covid healthcare in the early phases of the Covid-19 pandemic: Validated, diverted and rejected candidacy. SSM - Qualitative Research in Health. 2023;3:100207. DOI: 10.1016/j.ssmqr.2022.100207
37. Moretti C, Collaro C, Terzoni C, Colucci G, Copelli M, Sarli L, et al. Dealing with uncertainty. A qualitative study on the illness' experience in patients with Long-COVID in Italy. Acta Biomed. 2022;93(6):e2022349. DOI: 10.23750/abm.v93i6.14059
38. O'Brien KK, Brown DA, McDuff K, St Clair-Sullivan N, Solomon P, Carusone SC, et al. Conceptualising the episodic nature of disability among adults living with Long COVID: a qualitative study. Bmj Global Health. 2023;8(3). DOI: 10.1136/bmjgh-2022-011276
39. Rushforth A, Ladds E, Wieringa S, Taylor S, Husain L, Greenhalgh T. Long Covid – The illness narratives. Social Science & Medicine. 2021;286:114326. DOI: 10.1016/j.socscimed.2021.114326
40. Russell D, Spence NJ, Chase JD, Schwartz T, Tumminello CM, Bouldin E. Support amid uncertainty: Long COVID illness experiences and the role of online communities. SSM Qual Res Health. 2022;2:100177. DOI: 10.1016/j.ssmqr.2022.100177
41. Schmachtenberg T, Muller F, Kranz J, Dragaqina A, Wegener G, Konigs G, et al. How do long COVID patients perceive their current life situation and occupational perspective? Results of a qualitative interview study in Germany. Frontiers in Public Health. 2023;11. DOI: 10.3389/fpubh.2023.1155193
42. Taylor AK, Kingstone T, Briggs TA, O'Donnell CA, Atherton H, Blane DN, et al. 'Reluctant pioneer': A qualitative study of doctors' experiences as patients with long COVID. Health Expectations. 2021;24(3):833-42. DOI: 10.1111/hex.13223
43. Wurz A, Culos-Reed SN, Franklin K, DeMars J, Wrightson JG, Twomey R. "I feel like my body is broken": exploring the experiences of people living with long COVID. Quality of Life Research. 2022;31(12):3339-54. DOI: 10.1007/s11136-022-03176-1
44. O'Hare AM, Vig EK, Iwashyna TJ, Fox A, Taylor JS, Viglianti EM, et al. Complexity and Challenges of the Clinical Diagnosis and Management of Long COVID. Jama Network Open. 2022;5(11). DOI: 10.1001/jamanetworkopen.2022.40332
45. Mattingly C, Garro L, editors. Narrative and the Cultural Construction of Illness and Healing: University of California Press; 2001.

For peer review only

46. Rhodes T, Lancaster K. Evidence-making interventions in health: A conceptual framing. Social Science & Medicine. 2019;238:112488. DOI: 10.1016/j.socscimed.2019.112488
47. Cheshire A, Ridge D, Clark LV, White PD. Sick of the Sick Role: Narratives of What "Recovery" Means to People With CFS/ME. Qualitative Health Research. 2021;31(2):298-308. DOI: 10.1177/1049732320969395
48. Brown B, Huszar K, Chapman R. 'Betwixt and between'; liminality in recovery stories from people with myalgic encephalomyelitis (ME) or chronic fatigue syndrome (CFS). Sociology of Health & Illness. 2017;39(5):696-710. DOI: 10.1111/1467-9566.12546
49. Nettleton S. 'I just want permission to be ill': Towards a sociology of medically unexplained symptoms. Social Science & Medicine. 2006;62(5):1167-78. DOI: 10.1016/j.socscimed.2005.07.030
50. Jutel A. Sociology of diagnosis: a preliminary review. Sociology of Health & Illness. 2009;31(2):278-99. DOI: 10.1111/j.1467-9566.2008.01152.x
51. Clare E. Brilliant Imperfection: Grappling with Cure: Duke University Press; 2017.
52. Bailey CW. On the Impossible: Disability Studies, Queer Theory, and the Surviving Crip. Disability Studies Quarterly. 2019;39(4). DOI: 10.18061/dsq.v39i4.6580
53. Lancaster K, Duke K, Ritter A. Producing the 'problem of drugs': A cross national-comparison of 'recovery' discourse in two Australian and British reports. International Journal of Drug Policy. 2015;26(7):617-25. DOI: 10.1016/j.drugpo.2015.04.006
54. LaMarre A, Rice C. Recovering Uncertainty: Exploring Eating Disorder Recovery in Context. Culture, Medicine, and Psychiatry. 2021;45(4):706-26. DOI: 10.1007/s11013-020-09700-7
55. Vitellone N, Theodoropoulou L, Manchot M. Recovery as a minor practice. International Journal of Drug Policy. 2022;107:103618. DOI: 10.1016/j.drugpo.2022.103618

For peer review only

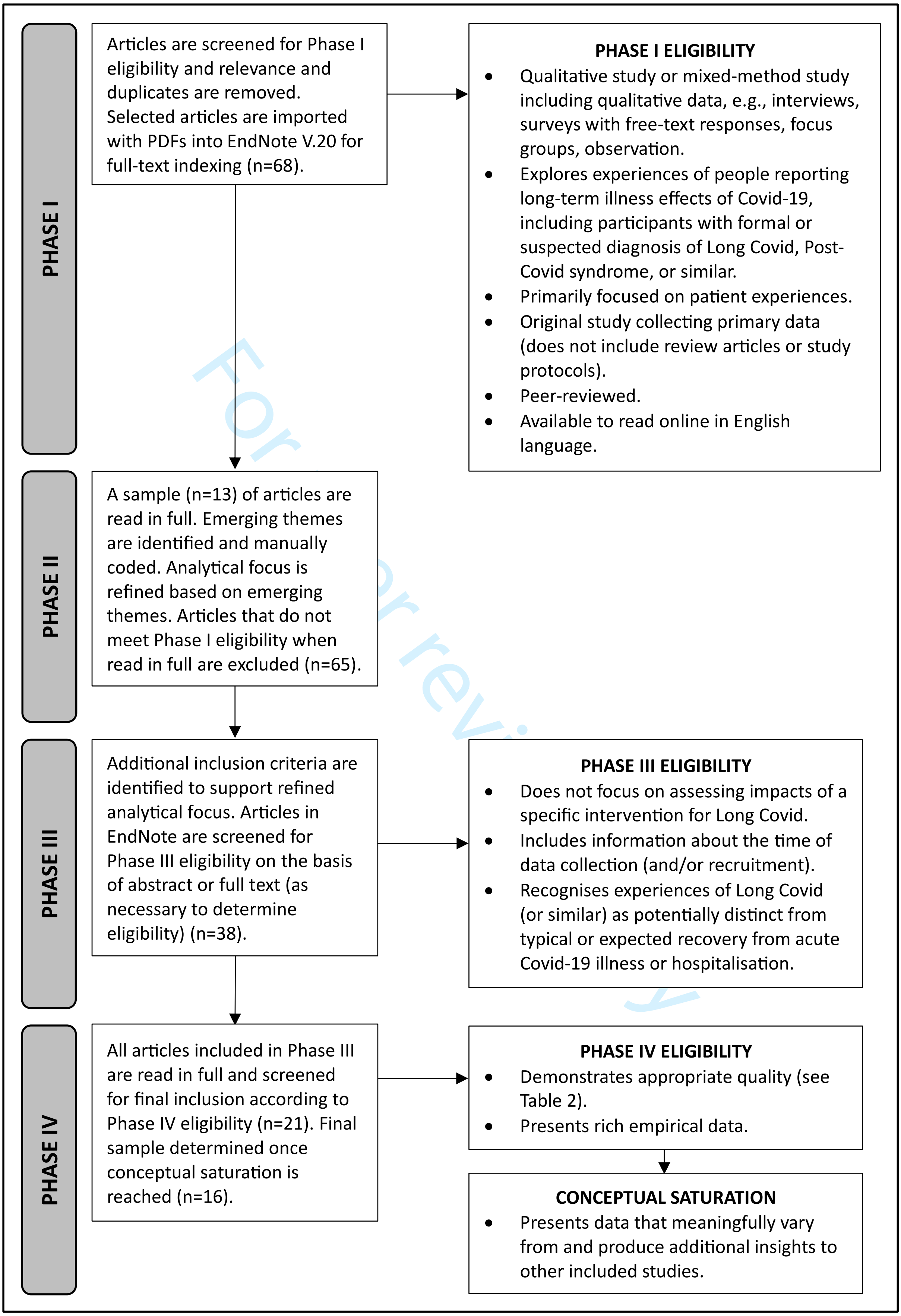

For peer review only

For peer review only - http://bmjopen.bmj.com/site/about/guidelines.xhtml

Supplemental Table 1: Database search queries

Database	Search Query	Results
PubMed	((postacute[Title/Abstract]) OR ("post acute"[Title/Abstract]) OR (pasc[Title/Abstract]) OR (persistent symptoms[Title/Abstract]) OR (prolonged symptoms[Title/Abstract]) OR ("long covid"[Title/Abstract]) OR (post covid syndrome[Title/Abstract])) AND (qualitative[Title/Abstract]) AND (covid[Title/Abstract])	144
Web of Science	(AB=(postacute) OR AB=("post acute") OR AB=(pasc) OR AB=(persistent symptoms) OR AB=(prolonged symptoms) OR AB=("long covid") OR AB=(post covid syndrome)) AND AB=(qualitative) AND AB=(covid)	187

**Supplemental Table 2:** Quality appraisal prompts
• Are the objectives of the research clearly stated?<br>• Is the research design clearly described and appropriate?<br>• Are data collection methods transparent?<br>• Are data analysis methods clearly described and appropriate?<br>• Are there sufficient data to support the authors' interpretations?

Quality appraisal prompts citation:

Harrison M, Rhodes T, Lancaster K. How do care environments shape healthcare? A synthesis of qualitative studies among healthcare workers during the COVID-19 pandemic. *BMJ Open*. 2022;12(9):e063867. DOI: 10.1136/bmjopen-2022-063867

For peer review only

ENTREQ Checklist

No	Item	Guide and description	Location where reported
**1**	Aim	State the research question the synthesis addresses.	Abstract; Introduction; Methodology
**2**	Synthesis methodology	Identify the synthesis methodology or theoretical framework which underpins the synthesis, and describe the rationale for choice of methodology *(e.g. meta-ethnography, thematic synthesis, critical interpretive synthesis, grounded theory synthesis, realist synthesis, meta-aggregation, meta-study, framework synthesis).*	Title; Abstract; Introduction; Methodology
**3**	Approach to searching	Indicate whether the search was pre-planned (*comprehensive search strategies to seek all available studies)* or iterative (*to seek all available concepts until they theoretical saturation is achieved*).	Methodology; Search Strategy
**4**	Inclusion criteria	Specify the inclusion/exclusion criteria *(e.g. in terms of population, language, year limits, type of publication, study type).*	Methodology (inclusion); Figure 1
**5**	Data sources	Describe the information sources used (e.g. *electronic databases (MEDLINE, EMBASE, CINAHL, psycINFO, Econlit), grey literature databases (digital thesis, policy reports), relevant organisational websites, experts, information specialists, generic web searches (Google Scholar) hand searching, reference lists)* and when the searches conducted; provide the rationale for using the data sources.	Methodology (search strategy); Supplemental Table 1
**6**	Electronic Search strategy	Describe the literature search *(e.g. provide electronic search strategies with population terms, clinical or health topic terms, experiential or social phenomena related terms, filters for qualitative research, and search limits)*.	Methodology (search strategy); Supplemental Table 1

For peer review only

No	Item	Guide and description	Location where reported
**7**	Study screening methods	Describe the process of study screening and sifting *(e.g. title, abstract and full text review, number of independent reviewers who screened studies).*	Methodology (inclusion); Figure 1
**8**	Study characteristics	Present the characteristics of the included studies *(e.g. year of publication, country, population, number of participants, data collection, methodology, analysis, research questions).*	Methodology (inclusion); Table 1
**9**	Study selection results	Identify the number of studies screened and provide reasons for study exclusion *(e,g, for comprehensive searching, provide numbers of studies screened and reasons for exclusion indicated in a figure/flowchart; for iterative searching describe reasons for study exclusion and inclusion based on modifications t the research question and/or contribution to theory development).*	Figure 1
**10**	Rationale for appraisal	Describe the rationale and approach used to appraise the included studies or selected findings *(e.g. assessment of conduct (validity and robustness), assessment of reporting (transparency), assessment of content and utility of the findings).*	Methodology (inclusion); Supplemental Table 2
**11**	Appraisal items	State the tools, frameworks and criteria used to appraise the studies or selected findings *(e.g. Existing tools: CASP, QARI, COREQ, Mays and Pope* [25]*; reviewer developed tools; describe the domains assessed: research team, study design, data analysis and interpretations, reporting).*	Methodology (inclusion); Supplemental Table 2
**12**	Appraisal process	Indicate whether the appraisal was conducted independently by more than one reviewer and if consensus was required.	Author contributions
**13**	Appraisal results	Present results of the quality assessment and indicate which articles, if any, were weighted/excluded based on the assessment and give the rationale.	Figure 1

For peer review only - http://bmjopen.bmj.com/site/about/guidelines.xhtml

No	Item	Guide and description	Location where reported
**14**	Data extraction	Indicate which sections of the primary studies were analysed and how were the data extracted from the primary studies? *(e.g. all text under the headings "results /conclusions" were extracted electronically and entered into a computer software).*	Methodology (data extraction and analysis)
**15**	Software	State the computer software used, if any.	Figure 1
**16**	Number of reviewers	Identify who was involved in coding and analysis.	Author contributions
**17**	Coding	Describe the process for coding of data *(e.g. line by line coding to search for concepts).*	Methodology (data extraction and analysis)
**18**	Study comparison	Describe how were comparisons made within and across studies *(e.g. subsequent studies were coded into pre-existing concepts, and new concepts were created when deemed necessary).*	Methodology (data extraction and analysis)
**19**	Derivation of themes	Explain whether the process of deriving the themes or constructs was inductive or deductive.	Methodology (data extraction and analysis)
**20**	Quotations	Provide quotations from the primary studies to illustrate themes/constructs, and identify whether the quotations were participant quotations of the author's interpretation.	Findings; Table 2
**21**	Synthesis output	Present rich, compelling and useful results that go beyond a summary of the primary studies (e.g. *new interpretation, models of evidence, conceptual models, analytical framework, development of a new theory or construct).*	Findings; Discussion; Conclusion

Checklist citation:

For peer review only

Tong, A., Flemming, K., McInnes, E. et al. Enhancing transparency in reporting the synthesis of qualitative research: ENTREQ. *BMC Med Res Methodol.* 2012;**12**(181). DOI: 10.1186/1471-2288-12-181

For peer review only

The constitution of Long Covid illness, patienthood, and recovery: A critical synthesis of qualitative studies

Journal:	*BMJ Open*
Manuscript ID	bmjopen-2023-083340.R1
Article Type:	Original research
Date Submitted by the Author:	28-Feb-2024
Complete List of Authors:	Harrison, Mia; University of New South Wales, Centre for Social Research in Health<br>Rhodes, Tim; London School of Hygiene & Tropical Medicine; University of New South Wales, Centre for Social Research in Health<br>Lancaster, Kari; University of New South Wales, Centre for Social Research in Health; Goldsmiths University of London,
**Primary Subject Heading**:	Qualitative research
Secondary Subject Heading:	Infectious diseases, Patient-centred medicine, Rehabilitation medicine, Sociology, General practice / Family practice
Keywords:	Post-Acute COVID-19 Syndrome, Patients, QUALITATIVE RESEARCH, COVID-19, Patient-Centered Care

I, the Submitting Author has the right to grant and does grant on behalf of all authors of the Work (as defined in the below author licence), an exclusive licence and/or a non-exclusive licence for contributions from authors who are: i) UK Crown employees; ii) where BMJ has agreed a CC-BY licence shall apply, and/or iii) in accordance with the terms applicable for US Federal Government officers or employees acting as part of their official duties; on a worldwide, perpetual, irrevocable, royalty-free basis to BMJ Publishing Group Ltd ("BMJ") its licensees and where the relevant Journal is co-owned by BMJ to the co-owners of the Journal, to publish the Work in this journal and any other BMJ products and to exploit all rights, as set out in our licence.

The Submitting Author accepts and understands that any supply made under these terms is made by BMJ to the Submitting Author unless you are acting as an employee on behalf of your employer or a postgraduate student of an affiliated institution which is paying any applicable article publishing charge ("APC") for Open Access articles. Where the Submitting Author wishes to make the Work available on an Open Access basis (and intends to pay the relevant APC), the terms of reuse of such Open Access shall be governed by a Creative Commons licence – details of these licences and which Creative Commons licence will apply to this Work are set out in our licence referred to above.

Other than as permitted in any relevant BMJ Author's Self Archiving Policies, I confirm this Work has not been accepted for publication elsewhere, is not being considered for publication elsewhere and does not duplicate material already published. I confirm all authors consent to publication of this Work and authorise the granting of this licence.

For peer review only

Title

The constitution of Long Covid illness, patienthood, and recovery: A critical synthesis of qualitative studies

Corresponding author

Mia Harrison

Centre for Social Research in Health, University of New South Wales, High St, Kensington, NSW 2052, Australia.

mia.harrison@unsw.edu.au

Author 1

Mia Harrison

1) Centre for Social Research in Health, University of New South Wales, Sydney, Australia

Author 2

Tim Rhodes

1) London School of Hygiene & Tropical Medicine, London, United Kingdom
2) Centre for Social Research in Health, University of New South Wales, Sydney, Australia

Author 3

Kari Lancaster

1) Centre for Social Research in Health, University of New South Wales, Sydney, Australia
2) Goldsmiths, University of London

Word Count

5373

For peer review only

Abstract

Objectives: To investigate lived experiences of Long Covid.

Design: Critical interpretive synthesis (CIS) of qualitative research.

Data sources: PubMed and Web of Science databases were searched 14 September 2023.

Eligibility criteria: Original peer-reviewed qualitative studies describing experiences of Long Covid were eligible for inclusion.

Data extraction and synthesis: We used established qualitative synthesis methods to search, screen, and manually code included studies. Critical interpretation methods were used to analyse data and develop synthetic constructs.

Results: 68 articles were identified in the first phase of sampling with 16 studies and 879 participants included in the final synthesis. Analysis of these studies was organised into three thematic constructions of Long Covid: 1) the illness; 2) the patient; 3) recovery. Long Covid was diversely characterised across study approaches, designs, and findings, but was underpinned by shared diagnostic logics, which shaped the identification and measurement of symptoms. The boundaries between different constitutions of Long Covid in qualitative accounts of illness experience were often imprecise. Slippages between different definitions of Long Covid had implications for patient experiences in relation to diagnosis, help-seeking and care, and expectations of recovery.

Conclusions: Long Covid is a site of multiple and diverse qualitative interpretation. Accounts of lived experience emphasise constitutions of illness, patienthood, and recovery as situated and emergent. The ongoing context-based negotiation of Long Covid is a defining qualitative feature of the condition. Approaches to researching, diagnosing, and developing health interventions must be as adaptive as the varieties of Long Covid lived experience.

Keywords

Qualitative synthesis; Long Covid; Lived experience; Patienthood; Recovery

Article Summary

Strengths and limitations of this study

- This study employed a critical interpretive synthesis method for review of literature, which enabled the development of new insights about the constitution of Long Covid, extending the goals of the included studies.
- This study developed synthetic constructs that allowed for analysis of primary evidence and second order analyses, which enabled critical reflection on how the methods and approaches of included studies shaped how Long Covid could be understood.
- Included studies were primarily recruited from Long Covid support groups or were developed as qualitative adjuncts to existing Long Covid cohort studies.
- Included studies sampled primarily from UK and US contexts.

For peer review only

- Included studies primarily used interview and focus group methods.

For peer review only

Introduction

This paper synthesises qualitative research describing lived experiences of Long Covid. Long Covid is generally characterised in the literature as an uncertain, emerging, and complex illness or health condition consisting of a range of possible symptoms that persist following Covid-19 infection [1-3]. As research and awareness of the long-term health effects of Covid-19 have increased, understandings of Long Covid have broadened to encompass diverse illness effects, care needs, and trajectories of illness and recovery [4, 5]. This has led to the rapid development of clinical knowledge and practice guidelines (coproduced via clinician and patient collaboration) that account for variations and complex presentations of Long Covid [6]. Yet the classification of Long Covid and expectations of post-Covid-19 recovery remain a subject of contestation and social and medical critique [4, 5, 7].

Systematic reviews of Long Covid studies have investigated characteristics, frequency, and heterogeneity of individual Long Covid symptoms (e.g., fatigue, cough, chest pain, memory loss, dyspnea, depression) [1, 8-10]. These reviews have also noted temporal information (e.g., average symptom duration), with one study systematically mapping the temporal persistence of individual symptoms post-infection at 12 weeks and 6 months [10]. Other reviews have synthesised studies focused on specific symptoms into categories, such as affected bodily systems (e.g., cardiopulmonary, respiratory, multisystem) [11-13] or affected areas of health (i.e., physical, mental, social) [14]. One review specifically compared clinical presentation and symptomology of Long Covid and myalgic encephalomyelitis/chronic fatigue syndrome (ME/CFS) [15].

Reviews have also investigated Long Covid interventions, management, and implications for clinical practice [2, 12, 16, 17]. One review grouped post-Covid symptoms into proposed subtypes (e.g., non-severe Covid-19 multi-organ sequelae, pulmonary fibrosis sequelae, ME/CFS) and identified potential interventions for each subtype [18].

Previous review work has tended to focus on clinical constitutions of Long Covid, with inadequate attention to how Long Covid is experienced and shaped by social relations and contexts. Reviews focusing on qualitative evidence have made greater contributions in such areas, though these are limited in number and scope. One qualitative review of 4 studies focused on the specific experiences of living with pulmonary sequalae and its implications for nursing practice [2]. Other reviews of qualitative research on living with Long Covid have synthesised the emotional and psychosocial impacts of Long Covid and patient experiences in accessing healthcare and resources [3, 19, 20].

One scoping review took a critical sociological approach, synthesising 93 studies (including qualitative studies) to explore clinical definitions and diagnostic constructions of Long Covid [5]. This review focused on the consequences of broadening definitions of 'Long Covid,' and argued that overly generous diagnostic boundaries can produce unintended forms of medical ignorance and uncertainty [5]. This analysis contributes to an emerging body of critical literature investigating how Long Covid has come to be classified across medical and social domains [4, 7, 21].

For peer review only - http://bmjopen.bmj.com/site/about/guidelines.xhtml

Our paper responds to and extends upon critiques regarding broad and fluctuating classifications of Long Covid and their implications for practice and policy [4, 5, 7, 21]. We develop a critical interpretive synthesis of qualitative research describing patient experiences and clinical interpretations of Long Covid. Our synthesis attends to both qualitative accounts of these experiences within the literature, and to how Long Covid becomes understood through the framing, methods, and analysis of qualitative empirical studies themselves. This enables us to attend to Long Covid in relation with three separate but interconnected concerns: Long Covid as an illness or diagnosis; Long Covid patients and their care needs; and experiences of (Long) Covid recovery.

We approach Long Covid as an illness category that is relationally constituted along with emerging knowledges and practices of scientists, clinicians, patients, and other stakeholders [22]. Making sense of Long Covid is thus "a matter […] of negotiation," in which we eschew any goal of a single and definitive description of Long Covid [23]. Our synthesis instead develops tools for understanding the experiences, stakes, and implications of Long Covid in different health and social situations, and for patients themselves.

Methodology

This paper uses critical interpretive synthesis (CIS) methods to review qualitative studies on patient experiences of Long Covid illness, recovery, and care. CIS methods prioritise sampling of rich and diverse data and are preferable in cases where the goal of the review is to generate theoretical insights and contributions beyond the findings of the original studies [24-26]. This requires critical, iterative, and reflexive approaches to developing a research question and undertaking sampling and analysis, rather than the systematic strategies utilised in many other review methodologies [25, 27]. We outline the steps of our methods below, which build upon an existing adapted approach to CIS methods [28].

Search strategy

We employed a combination of search strategies to identify literature for potential inclusion. First, we conducted searches of PubMed and Web of Science databases 14 September 2023 using analogous search queries (Supplemental Table 1). As CIS methods do not require a systematically generated sample of literature, additional manual searches and citation chaining methods were periodically conducted (until 15 December 2023) to identify other relevant studies for consideration [28, 29]. These manual search strategies did not yield additional articles for inclusion.

Inclusion

We developed four phases of sampling, adapted from an existing CIS approach [28]. These are presented in a simplified form in Figure 1; however, in practice these phases were not discrete or linear steps, but instead unfolded during the review process through reflexive, overlapping, and iterative practices of testing and analytical refinement. Eligibility for inclusion was kept intentionally broad in Phase I and was narrowed as we refined our

For peer review only - http://bmjopen.bmj.com/site/about/guidelines.xhtml

analytical focus, with additional criteria developed to support a higher overall interpretive value across the final sample [24]. Quality was appraised with a goal of maximising conceptual inclusion [24] using previously developed appraisal prompts [28] (Supplemental Table 2). Final inclusion was determined according to principles of conceptual saturation [24, 27], with studies included on the basis of contribution to theory development. Studies that did not produce any new insights from other included studies were excluded in Phase IV.

Our final sample included 16 studies and 879 participants with Long Covid or similar experiences (not accounting for potential duplication between studies), with most participants living in the UK or USA (Table 1). Primary study data was generated from these studies between July 2020 and May 2022. Most studies generated qualitative data through semi-structured interviews or focus groups (of samples ranging from 13 to 114 participants recruited via a combination of surveys, cohorts, social media, and clinical or community networks) [30-43] with two studies additionally generating visual data through participant illustration [39] and artist interpretation [42] methods. Other studies generated qualitative data via electronic health records (N=200) [39] and open-ended responses from a larger observational online survey (N=169) [44]. Studies used interpretive or descriptive thematic analysis methods, with half of included studies developing sociologically informed theoretical frameworks [31, 33, 36, 37, 39-42].

Table 1: Included studies

First author (year)	Country	Sample description (N)	Definition of Long Covid to determine study eligibility	Data collection period	Data description
Aghaei (2022) [30]	USA	Female long haulers (15)	Infected with Covid-19 and having experienced at least one Covid-19 symptom lasting four weeks or longer after a Covid-19 diagnosis	April to mid-June 2021	Interviews
Bergmans (2023) [31]	USA	Black patients with Long Covid (15)	Physical or mental health symptoms that lingered over one month after an acute Covid-19 infection	May to September 2021[1]	Interviews
Burton (2022) [32]	UK	People who self-reported Long Covid (21)	A positive swab test/antibody test or one or more commonly reported Covid-19 symptoms at illness onset (persistent cough, loss or change in taste/smell, high temperature); and experiencing one or more broader symptoms ≥3 weeks following the onset of their first symptoms	November 2020 to September 2021	Interviews
Callan (2022) [33]	UK	People with self-defined Long Covid and people from an online support group dedicated to Long Covid's	Not specified	October to November 2020	Focus groups

For peer review only - http://bmjopen.bmj.com/site/about/guidelines.xhtml

		neurocognitive effects (50)			
Chasco (2022) [34]	USA	Patients of a Midwestern academic hospital's post-Covid-19 clinic (15)	Persistent health concerns more than 3 months after SARS-CoV-2 infection	July to October 2021	Interviews
Cooper (2023) [35]	UK	People from a West London Long Covid clinical assessment centre (13)	Documented history of SARS-CoV-2 infection and reported persistent or new symptoms (> 12 weeks) following Covid-19	May to July 2021	Interviews
Fang (2023) [36]	UK	People self-identified as experiencing Long Covid symptoms (80)	Reported Covid-19-related symptoms for over 4 weeks, particularly focused on those who indicated having persistent symptoms for over 8 weeks	November 2021 to March 2022	Interviews
Maclean (2023) [37]	UK, USA, Netherlands, Canada, Australia	People with (Long) Covid (72)	Not specified	November 2020 to March 2022	Interviews
Moretti (2022) [38]	Italy	Women with Long Covid (17)	Experiencing Long Covid symptoms for at least three months following confirmed Covid-19 infection; having perceived a significant impact of Long Covid symptoms on quality of life; having consulted multiple healthcare professionals in order to receive a diagnosis	November to December 2021	Interviews
O'Brien (2023) [39]	Canada, USA, Ireland, UK	Adults who self-identified as living with Long Covid (40)	Signs and symptoms that develop during or following an infection consistent with Covid-19 that continue for 12 weeks or more and are not explained by an alternative diagnosis	December 2021 to May 2022	Interviews and participant visual illustrations
O'Hare (2022) [45]	USA	Veterans with Long Covid (200)	Documentation of a positive result on a polymerase chain reaction (PCR) test for SARS-CoV-2 and an ICD-10 diagnostic code for Long Covid	February to May 2022	Electronic health records
Ruthforth (2021) [40]	UK	People with Long Covid (114)	Not specified	May to October 2020	Interviews and focus groups
Russell (2022) [41]	USA	Self-identified long-haulers from online Covid-19 communities (20)	Long-term symptoms and effects from Covid-19 with one or more consultations with a healthcare professional about Covid-19 symptoms	March to April 2021	Interviews
Schmachtenberg (2023) [42]	Germany	Long Covid patients (25)	Symptoms that develop during or after an infection consistent with Covid-19, continue for more than 4 weeks, and are not explained by an alternative diagnosis	January to May 2022	Interviews and graphical artist interpretations
Taylor (2021) [43]	UK	Doctors with Long Covid (13)	Not specified	July to August 2020	Interviews

For peer review only - http://bmjopen.bmj.com/site/about/guidelines.xhtml

Wurz (2022) [44]	International (92% from UK, USA, or Canada)	Adults living with Long Covid (169)	Currently experiencing long-term symptoms due to Covid-19 (at least 4 weeks since the acute illness or positive Covid-19 test, with symptoms not predating the acute illness); and having tested positive for Covid-19, or with probable infection (based on an illness mimicking the acute phase of Covid-19, having close contact with a confirmed case, or being linked with an outbreak), in line with the clinical case definition for post-Covid-19 condition	February to April 2021	Open-ended responses in online survey
[1] This is the period of participant recruitment, as the period of data collection was not specified.					

Data extraction and analysis

MH identified and manually coded emerging themes in Phase II of sampling (Figure 1) and developed a conceptual argument in collaboration with TR and KL [22]. All three authors have expertise in critical social science approaches to analysis of qualitative data in health, which informed our theoretical approach to theme generation. These early themes guided the development of additional eligibility criteria, which were used to refine the final sample based on contribution to the conceptual goal of the synthesis.

MH led development of synthetic constructs to organise the conceptual argument. These constructs were refined in collaboration between the authors through our reflexive and iterative sampling strategy (Figure 1). Synthetic constructs allow for both underlying evidence and second order analyses in included studies to be assembled, critically analysed, and transformed, with a goal of developing new conceptual arguments and analytical insights [24]. We thus approached data analysis of included studies at two orders: analysis of the primary data presented in the articles and analysis of the interpretations of primary data developed by the authors of the studies [24, 28].

Following Dixon-Woods et al. [24], our analyses of included studies develop a synthesising argument that is grounded in the data yet extends upon the findings of included studies. This enables us to develop new insights and make sense of the research in a novel and explanatory way. Our synthesising argument assembles both our synthetic constructs and second order interpretations of data presented by included studies. This is an established approach to CIS methods [24, 28] but is especially valuable for our analysis of Long Covid. A key concern has been how Long Covid is studied and understood by academics, clinicians, patients, and activists [4, 5, 7, 21]. Our critical interpretation of study authors' interpretations of Long Covid data is therefore not only an appropriate approach to CIS analysis; it also constitutes a crucial line of inquiry for investigating how Long Covid has become known, diagnosed, experienced, researched, and cared for as an emerging, uncertain, and contested health condition.

For peer review only - http://bmjopen.bmj.com/site/about/guidelines.xhtml

Patient and Public Involvement

There was no patient or public involvement in this study.

Findings

Our synthesis investigates lived experiences of Long Covid. We organise our analysis into three thematic constructions of Long Covid: the illness, the patient, and recovery. Table 2 presents illustrative participant extracts from included studies, organised by theme and subtheme.

Table 2: Illustrative participant extracts from studies

Theme	Subtheme	Example Quote
The illness	Complex symptom presentation	"I had an odd rash for quite a while it kept coming and going ... very itchy cough ... very mild asthma […] I just kept putting it down to grief until a couple of months in a friend said, 'Look do you think this could be Covid?'" [40]<br><br>"The biggest challenge is not knowing when [the episodes are] going to happen, how long they're going to last or how much I can get away with before causing it, if I am truly the cause of one of these episodes." [39]
	Symptoms as inherently strange	"It's been rather mysterious […] and it doesn't feel like anything else, it's bizarre." [43]<br><br>"The fatigue is not exactly tiredness as in sleepiness. It is fatigue as in how a healthy person would feel the day after they ran a marathon." [44].
	Hiding implausible symptoms	"I'd often get a heart rate of 130, just randomly watching TV. And avoided telling my GP about that. […] I didn't want to admit it because I think I was worried about it being dismissed as anxiety." [43]<br><br>"I thought had gotten better, so you know, the phantom smells, the tinnitus, I'd had some strange nerve pain, like a poker in my ear, really bizarre, but it was very, very painful. Strange kind of scalp sensations, as well, but all, all such vague and strange things, I didn't even mention them to, to my GP, because I thought she might think I was completely mad." [37]
	Fuzzy symptom categories and ambiguous causation chains	"I have constant headaches, that is true, but I don't know if that is because of COVID or just because I'm stressed, it is psychological." [35]<br><br>"She also relates a history of asthma and chronic cough. Difficult at this time to separate out post COVID recovery from potential underlying asthma." [45]
The patient	Thresholds of symptom severity for patienthood	"I feel like it probably doesn't apply to me, because I feel like my symptoms, or whatever I have left, is not as severe as what other people are going through." [35]

For peer review only - http://bmjopen.bmj.com/site/about/guidelines.xhtml

		"I don't want to put any pressure on [the] health service. [It] is quite awful to come and approach a GP and say, 'I'm really tired.'" [37]
	Disrupted daily life as evidence of patienthood	"I used to go out dancing twice a week [...] and you think, well, I couldn't even get up, let alone go dancing twice a week... You reach a point where you don't actually remember being well." [36]<br>"I had COVID and five months later, I'm still not better. [...] I can't look after my children for long periods of time, or in the initial stages at all, and that created huge problems." [43]
	Health professionals as authenticators of patienthood	"I have come across a few doctors where I've felt they've made me feel a bit stupid when I'm worrying about certain things ... So yes, there's that worrying that people just think you're making it up." [32]<br>"[The doctor] listened to absolutely everything I had to say, every weird symptom that I thought was unrelated. You know, he listened to everything and then would tell me, 'Nope, that's all part of it.'" [37]
	Managing care through self-experimentatio n	"I have gone all out on the quackery, which I never thought I would because I've never been that person. I've had acupuncture a few times. I am under a dietician and I'm taking all sorts of weird and wonderful supplements." [43]<br>"I even let my daughter and I let my friend, my mom, wear my Apple Watch for a day. Like, 'Let me see if [the high heart rate notification] goes off for you.' If it's not going off for you, then why is it going off for me?" [31]
Recover y	Illness and recovery as temporary	"You don't feel good, but you have to get to work, and then you have kids and family. [...] You are expected to do [it] all." [30]<br>"I also had two colleagues who complained about me to the staff council, along the lines of 'I always have to cover for her because she is sick.'" [42]
	Non-linear recovery trajectories	"I liken it to a rollercoaster ride and you're like up and down... there are good days and bad days.... my symptoms are still there but it kind of goes down." [39]<br>"You feel like shit for a few days [with Covid-19], you start to feel a little bit better, then you feel like shit again [...] it's about a whole new experience of illness. [...] You come to doubt your knowledge of illness and recovery." [37]
	Illness/wellness binaries	"In March, we thought COVID was something that would either kill me in three weeks or I'd get over it ... It was May when I started having long-term symptoms. [...] Am I the only one going through this or are there others?" [41]<br>"It's like when somebody has a stroke, and they get partially paralysed. [...] They might regain partial control, like learn to walk again, but their arm might not ever be working again." [41]

For peer review only

	Indeterminacy of acute/Long Covid recovery	"The Long Covid name to me, feels like you've still got COVID; it's like a long version of it. […] I always say post COVID, personally, just because usually when talking about things like post-op, you would say, not long-op." [35]<br>"It's a bit wishy-washy, to me anyway, but also, I think everyone is feeling that they've got slightly different as well. […] It's a bit of a broad term, isn't it?" [35]
	Restorative versus chronic models of illness	"I would rather [the term Long Covid] than say, chronic COVID, that would be a lot more worrying." [35]<br>"I consider myself disabled 24/7. […] I'm always at this minimal state, […] it's not like I go from 'oh today I can do all these things.' No, there's no day like that." [39]

For peer review only

The illness

The first question that studies on Long Covid have addressed is: 'what is Long Covid'? All but two of the included studies were published in 2022 or 2023, yet the complexity of this question remains a concern. Studies defined Long Covid using clinical guidelines and criteria (such as those of the World Health Organization and the UK National Institute for Health and Care Excellence) [32, 39, 42, 43], as well as on the basis of self-identification, noting the role of patient advocacy in the naming of Long Covid [33, 35-37, 40, 41, 44, 45]. The term Long Covid was generally used, though studies also acknowledged other labels such as "post-Covid syndrome," "Post-Acute Sequelae of Covid-19 (PASC)," and "long haulers" [30, 32-35, 38-45]. One study explicitly distinguished terms, using "long COVID-19" to describe "the lived patient experience," and "post-COVID-19 syndrome" to describe "the medically diagnosed condition" [33].

These working definitions of Long Covid informed participant inclusion criteria, which varied across the studies (Table 1). Most studies identified a minimum number of weeks that participants must have experienced persistent or new symptoms after acute illness (usually 4 or 12 weeks) [30-32, 34-36, 38, 39, 42, 44]. Some also stated preference for confirmed SARS-CoV-2 infection, such as through a positive test or a diagnosis from a clinician [32, 35, 44]. One study required a formal Long Covid diagnosis from a clinician [45]. A handful of studies determined eligibility through self-identification or did not define Long Covid in their reported eligibility criteria [33, 37, 40, 43].

All studies described Long Covid as a complex condition consisting of many and varied symptoms. While none of the included studies identified specific symptoms or illness effects as requisites for (or not included within) Long Covid diagnoses, the symptomology and aetiology of Long Covid were primary concerns. All studies we reviewed described specific symptoms experienced by participants, with some studies additionally tracking how common particular symptoms were across the study sample [31, 32] or how long symptoms had persisted [31, 32, 34, 37, 39, 41, 44]. Symptoms tended to be described in generally

recognisable terms (such as “brain fog,” “fatigue,” “headaches,” “shortness of breath”), with a focus on how symptoms were managed and impacted on participants’ daily lives.

Yet in several studies, participants also emphasised the “strangeness” of their symptoms, suggesting that commonly used terms inadequately described their embodied experiences [37, 40, 41, 43, 44]. Recognising these “strange” symptoms in others allowed people with various illness experiences to collectively identify with the terminology of Long Covid. When these diverse presentations were not widely recognised or accepted (especially early in the pandemic), some participants avoided disclosing symptoms they considered to be “implausible” [37, 43]. Other participants made sense of complex Long Covid effects through comparisons to health conditions with better understood or more widely accepted biological mechanisms, such as stroke or traumatic brain injury [33, 35, 41, 43].

Several studies emphasised that symptoms should not have predated Covid-19 infection or be explained by “alternative diagnoses” [39, 42, 44, 45]. Yet in practice, the presentation of Long Covid symptoms was often fuzzy and revealed slippages between descriptions of the symptoms of Long Covid and its extended effects. This was particularly common with psychological and mental health symptoms, with some studies describing symptoms such as anxiety and depression as direct manifestations of Long Covid, and others framing mental health impacts as part of a more complex chain of illness effects, for instance as a consequence of fatigue [30-32, 34, 35, 38, 44]. These ambiguous causation chains also intersected with the broader pandemic context [35]. In cases where participants had a pre-existing disability or health condition, experiences of Long Covid were sometimes described as an “exacerbation” of these conditions, further complicating the actualisation of Long Covid as a distinct and traceable illness [31, 35, 39, 45].

The patient

Because of the complexity and diversity of Long Covid symptoms, another challenge of characterising Long Covid was determining who counts as a Long Covid ‘patient’ (and in what situations). A priori study eligibility criteria inevitably stabilised the constitution of the Long Covid patient to some extent; yet the Long Covid patient was still heterogeneously defined in studies. For example, some participants referenced an imagined threshold of severity at which a person becomes ‘sick enough’ to be considered a ‘Long Covid patient’ (as defined by themselves or by healthcare professionals) [35, 41]. Overall, however, the ‘patient’ of Long Covid was presented with two defining features: symptoms that brought about *disruption* in daily life; and a need for *care* as a consequence of symptoms.

Studies generally evidenced “disruption” in daily life in relation to the capacity of participants to maintain an active social life [30, 32, 36, 38, 39, 42, 44], return to work [30, 32-34, 36, 38, 41-44], engage in physical activity and exercise [30-32, 34-36, 42, 44], or carry out domestic tasks [30, 32, 34, 36, 37, 39, 42, 44]. For example, “fatigue” was described in several studies in terms of participants having insufficient stamina to play the sports they enjoyed prior to Covid [31, 32, 36, 42, 44]. The boundaries of these illness effects were fuzzy and complex, with Long Covid’s disruptiveness intersecting with multiple other social and

For peer review only - http://bmjopen.bmj.com/site/about/guidelines.xhtml

environmental factors; for example, experiences of patienthood might impede the assumed caring responsibilities of motherhood [30, 31].

Many studies framed experiences of seeking care for Long Covid in relation with other experiences of “chronic illness,” “hidden disability,” and “contested illness,” including through comparisons to ME/CFS, HIV-related illnesses, or fibromyalgia [33, 34, 36-41, 43-45]. Management of Long Covid via formal health services was described as “fragmented,” insufficiently resourced, “poorly coordinated,” and lacking relevant knowledge or treatment options [32, 37, 38, 40, 41, 43, 45]. Participants expressed frustration at being expected to develop their own treatment plans and insufficient or ambiguous diagnostic and monitoring technologies for “making sense” of their Long Covid illness experience [37-40, 43]. Participants also reported difficulty in understanding processes for securing referrals to appropriate specialists [32, 37, 40], especially if neurological issues made it harder for them to advocate for themselves and explain their experiences [33]. Other barriers to accessing services included requiring formal diagnoses or positive Covid-19 tests [32, 37, 41], and changing eligibility criteria [32]. Some participants also described feelings of “guilt” for accessing an under-resourced health system, especially when people were dying from Covid-19 [37, 43].

Another care-related concern described in studies was in relation to the diagnostic category of Long Covid. Studies described experiences of medical “gaslighting” (especially earlier in the pandemic), where participants had to try to “prove” the reality of their illness and felt that their experiences were often “minimised” or “dismissed as anxiety” [31-33, 36-38, 40, 41, 43]. Participants emphasised the importance of having “weird” symptoms “validated” by medical professional as part of Long Covid and described this validation as a form of care in itself [33, 35, 37, 40, 43]. Participants also reported mixed experiences of care and support from family and friends, noting similar challenges to that of formal care seeking such as insufficient knowledge and resources, or “disbelief” [30, 31, 34, 36, 38, 40]. For example, some participants found initial support from friends and family became strained over time [32], especially when symptoms were “invisible” or “unpredictable” [39].

Across studies, care was actualised via “self-management,” self-investigation, and “hypothesising” about Long Covid illness. For instance, some studies described management of Long Covid through processes of embodied experimentation, such as through trying different diets, pharmacological interventions, vitamins and supplements, or alternative therapies [31, 33, 35, 38, 43, 44]. Many participants described managing their illness as a series of “trade-offs” or “compromises” where they worked out what they had the energy to do each day [30, 31, 33, 35, 39, 40, 44]. These experiments required participants to monitor their bodies to determine what was working or what could be triggering particular illness effects. Several participants also engaged in collective “sense-making” and knowledge generation via online Long Covid communities [31, 32, 37, 40, 41]. While these communities provided practical support and validation, participants in one study highlighted the potential dangers in these cultures of knowledge sharing, which could include “unverified medical advice” [41].

For peer review only - http://bmjopen.bmj.com/site/about/guidelines.xhtml

Recovery

Studies presented the experience of Long Covid recovery as marked by "uncertainty" [31-33, 37-39, 41, 45]. While some participants described recovery as an incremental process towards becoming well again [33, 44], far more studies included participants who described recovery in terms of "episodic illness" or "disability" [33, 39, 40, 44]. In these cases, illness became "unpredictable" in nature and was marked by "relapses" or periods of worsened symptoms [32-35, 37, 39-41, 44, 45]. This episodic experience of Long Covid disrupted narratives of recovery as a "linear pathway" from 'sick' to 'better' [37, 39-41, 43, 45]. Participants who had experienced Long Covid earlier in the pandemic found this particularly troubling, as their lived experiences of Long Covid differed greatly from the public understanding of Covid-19 illness (where the danger was perceived to lie in whether or not the acute illness caused death) [41, 43]. Some participants also described an initial period of "recovery" following acute illness or periods of "partial recovery" from Long Covid, which troubled this trajectory further [35, 40].

The chronicity of recovery raised ontological questions about the relationship between acute illness and Long Covid. Long Covid was characterised by some participants as existing on a "continuum" from the time of acute illness, while others saw it as "fluctuating," and organised as "clusters" or "coming in waves" [35, 39, 40]. One study described conflicting participant perspectives as to whether Long Covid even existed as a discrete illness or condition; some participants instead described their experiences as simply a period of recovery from Covid-19, as one might expect following any flu-like illness [35]. Others described Long Covid as an especially significant experience of recovery that might not ever result in a full return to their previous health [41]. The separation between recovery from acute illness and Long Covid as a distinct condition was complicated further when relapses resulted in hospitalisation, which could produce additional illness events from which to recover [45].

The terminology of Long Covid also related to how recovery was characterised. For some participants, labels like "Long Covid" validated their experiences as distinct from other illnesses (such as acute Covid-19 or ME/CFS) and as a recognisable illness shared with other people [34, 35, 37, 38]. However, one study described some participants feeling as though the label implied that their experiences were part of the *same* illness as acute Covid-19 [35]. Other language rendered the chronicity and ontology of Long Covid similarly complex. For example, some participants identified as being "disabled" or having a "chronic" illness or health condition [34, 36, 39, 41, 44]. These terms designated Long Covid as a stable and ongoing condition, rather than something to be recovered from (or a process of recovery in and of itself), which was a shift in framing that some participants found distressing [35, 36].

These experiences all intersected with expectations of what recovery 'should' look like. Participants described social and professional pressures to "recover" and return to "normal" [30, 31, 34, 42]. Participants' "guilt" about being unable to perform their previous social roles was compounded when they additionally required care from their family or partner [30, 34, 42]. Some participants internalised these expectations and questioned their

For peer review only - http://bmjopen.bmj.com/site/about/guidelines.xhtml

own sense of what it means to recover from illness [37, 40, 41]. One study summed up the conflict between expectations of recovery and the lived experience of Long Covid illness as a tension between the roles of Long Covid patient and “Covid-19 survivor” [30].

Discussion

Our synthesis generates three intersecting analytical themes relating to the epistemic construction of Long Covid: the illness, the patient, and recovery. We find that the constitution of Long Covid across these themes produces different implications for the research, management, and lived experience of Long Covid. This resonates with other social science literature on the diverse constitution of Long Covid [4, 7, 21]. One sociological review, for instance, has cautioned that flexible approaches to describing and diagnosing Long Covid can produce challenges for developing pragmatic care information and targeted health interventions [5].

Our discussion responds to this critique by accentuating the importance of flexible *yet situated* approaches to understanding Long Covid. We call attention to the many ways that Long Covid has been constituted *in* and *by* studies and the effects of this constitutive work. This underscores the importance of reflecting on how the methods and approaches of qualitative studies themselves shape the understandings of Long Covid they produce [22]. We conclude by reflecting on the emergent fluidity and diversity of lived experience in qualitative accounts as defining characteristics of the Long Covid condition. In doing so, we highlight the need for adaptive strategies in diagnosis and care.

Reflections on study design

The design and methods of qualitative study constitute the object of Long Covid, and thus how Long Covid comes to be understood, in specific ways. Studies variably framed Long Covid as a diagnostic category, a lived experience of illness or recovery, a social identity, a medical object of research or care, a policy concern, and more. Studies also defined Long Covid in relation to health guidelines and/or accounts emerging through patient advocacy, which diversely characterised Long Covid; for example, Table 3 presents descriptors that were *applied to* and *demarcated from* definitions and accounts of Long Covid across the studies. Inconsistencies between these characterisations of Long Covid (as well as across lived experiences and clinical interpretations described within study data) were generally navigated by framing Long Covid as an “uncertain,” “contested,” or “emerging” illness [33, 35-38, 41, 43, 44].

Table 3: Descriptors used in Long Covid in studies

- Acute Covid-19 illness
- Recovery following acute Covid-19 illness
- Post-Covid Syndrome or a similar formal diagnosis
- Extended effects of acute Covid-19 illness, hospitalisation, and/or rehabilitation including due to being unable to exercise or being on a ventilator

For peer review only - http://bmjopen.bmj.com/site/about/guidelines.xhtml

- Covid-19 symptoms persisting for a specified minimum length of time following onset of Covid-19
- Symptoms with understood and verifiable underlying biological mechanisms
- A discrete list of recognised and validated Long Covid symptoms
- Symptoms of pre-existing illness or disability that are exacerbated following Covid-19 illness
- Mental illness or psychological distress
- Chronic illness or disability
- A syndrome or collective label for many different illness experiences
- Symptoms that cannot be explained by other diagnoses
- ME/CFS

The constitution of Long Covid within participant eligibility criteria similarly varied across study methods. Most studies recruited participants through Long Covid support groups or by publicly advertising the study, though some [34-36, 45] sampled from clinical settings or existing cohorts, which enabled researchers to identify eligible participants from a broader repository of health data. All recruitment strategies required participants, researchers, or clinicians to identify Long Covid patients, which meant that inclusion was shaped by evolving understandings and public awareness of Long Covid at the time of recruitment.

Future qualitative studies need to be more open in their iterative sampling and exploration so as not to close-off or narrow Long Covid as a site which is emergent in its experience. Different qualitative study designs delimit constitutions of Long Covid differently a priori, which can shape as well as potentially narrow the accounts of lived experience that are produced; furthermore, participant accounts can suggest a greater fluidity and diversity of experience than a priori study definitions of Long Covid imply. This points to the limits of narrow definitions of Long Covid at the outset of studies, and the need for study designs to remain attentive to the more open accounts of lived experience that studies may produce.

We suggest that researchers should carefully consider how Long Covid is defined and understood beyond clinical and biomedical understandings. Several studies in our synthesis designed eligibility criteria in terms of self-identification [33, 35-37, 40, 41, 44, 45]. However, there was limited reflection on what might influence individuals to feel included or excluded from illness categories, and how information about illness is accessed and understood by publics. Future study designs that utilise self-identification might therefore pursue multiple recruitment strategies that use different language and descriptions to invite diverse experiences of the illness, health, or care concern being studied. A defining characteristic of the condition of Long Covid is its inherent fluidity, diversity, and multiplicity, and thus it is important for future studies to avoid building into their designs a priori definitions of Long Covid that are overly narrow, given how this can shape inclusion, recruitment, and accounts of illness experience.

For peer review only

We also emphasise a more general need for greater transparency and reflection on how conceptualisations of Long Covid shape study design, recruitment, and analysis. We found, for instance, that even studies with highly specific inclusion criteria extrapolated their findings to more loosely defined and diverse accounts of Long Covid that emerged in their analysis. It is important that future studies make transparent how their conceptualisations of Long Covid shape the constitutions of Long Covid produced. Similarly, studies should ensure epistemological consistency in how they frame and conduct their analyses. A general observation is that qualitative research in this emergent field is relatively under-theorised.

Studies in our synthesis largely employed 'realist' and/or 'critical realist' approaches and conducted descriptive and/or interpretive thematic analyses of semi-structured interview data. A minority of synthesised studies adopted 'critical' and/or 'social constructionist' approaches [33, 36-38, 40-42], focused for instance, on how narratives 'enact' or 'perform' constitutions of Long Covid [33, 36, 37, 40, 41]. There is greater scope for critical sociological qualitative analyses in this field—in particular, analyses that treat narrative as a process of enactment for study, rather than simply a resource of lived experience description [22, 46]. The constitution of Long Covid as an emergent, situated, and negotiated lived embodiment emphasises the importance of qualitative designs that approach illness, patienthood, and recovery as matters of 'construction' and 'enactment' [22].

From Long Covid symptoms to Long Covid patients

Across our synthesis, Long Covid was framed as an inherently symptomatic illness experience. Studies we reviewed did not identify *specific* symptoms as proving or precluding a Long Covid diagnosis or experience. Instead, studies accentuated Long Covid as an open category that included symptoms felt to be persistent or recurring following acute Covid-19 infection, symptoms felt to be new and different, as well as symptoms which came and went over time and with varying degrees of severity. Studies used different minimum timeframes of recurring or ongoing symptoms as a condition of Long Covid, yet the 'long' and 'symptomatic' nature of Long Covid was consistently identified as a defining feature of the illness experience and without a clear end point.

Another defining feature across studies was the constitution of Long Covid as a disruption to the 'normal.' Studies noted, and some deployed in their inclusion criteria [39, 42, 44], Long Covid symptoms as unexplainable by alternative diagnoses. However, we found that otherwise familiar symptoms, including from existing medical or health conditions [31, 35, 39, 45], were made 'strange' in studies through complex symptom clusters, heightened illness effects, and unpredictable recovery trajectories that disrupted 'normal' life [37, 40, 41, 43, 44]. Furthermore, the boundaries between the effects of Long Covid as a biomedical illness and the complex social worlds of patients (which included experiencing a global pandemic) were often fuzzy and variably defined [30-32, 34, 35, 38, 39, 44, 45]. This fuzziness made responding to and caring for Long Covid (in formal and informal settings) difficult and could result in participants feeling they had been denied Long

For peer review only - http://bmjopen.bmj.com/site/about/guidelines.xhtml

Covid patienthood, especially when symptoms were excluded from diagnostic consideration [31-33, 36-41, 43].

These inconsistencies and complexities in Long Covid criteria have been subject to critique [4, 5]. Yet our findings suggest that many of the challenges related to more open and changeable constitutions of Long Covid emerge as a result of insufficiently situating this constitutive work. There is a need for researchers and care providers to embrace broader and more open understandings of Long Covid 'symptoms' and 'diagnosis' that reflect the diversity of experiences examined across qualitative studies—not to capture more people within a singular definition of the 'Long Covid patient' with standardised 'care' needs, but to acknowledge the complex reality of Long Covid experiences. For example, our synthesis shows that a Long Covid diagnostic label can provide validation and help individuals make sense of their illness experience even in the absence of clinical care pathways [31-33, 35, 37, 40, 41, 43]. This emphasises the clinical and social value of Long Covid diagnosis beyond its capacity to support linkage to care or treatment.

The object of Long Covid recovery

Like Long Covid patienthood, recovery is multiply interpreted across the studies we reviewed. Our synthesis suggests that non-linear trajectories of Long Covid recovery, where illness does not become progressively better over time but is instead marked by unexpected or relapsing symptoms, is experienced as a disruption of recovery itself [39-41, 43, 45]. This produces new health uncertainties when expectations of what Long Covid recovery 'should' look like fail to materialise [30, 31, 34, 37, 41-43]. Across studies, these effects were most noticeable for participants who had experienced Long Covid earlier in the pandemic when there was less knowledge about post-Covid-19 illness and recovery [32, 33, 37, 40, 41, 43].

The non-linearity of Long Covid experiences also challenged notions of 'full' recovery or 'partial' recovery. Some participants experienced Covid-19 recovery as a separate process to Long Covid (recovery) [35, 40] and others resisted assumptions of recovery entirely, describing themselves as disabled [34, 36, 39, 41, 44]. Here our synthesis resonates with existing research on experiences of chronic illness, which argues that so-called 'restoration' of bodily capacities following illness and disability is not always realistic or possible [47, 48]. Furthermore, while diagnostic labels can give patients "permission to be ill" [49, 50], our findings suggest that this social permission is predicated on an assumption that illness is temporary and that patients will soon resume 'normal' professional and social activities [30, 31, 34, 42]. Such conditional freedom from social obligations also mirrors broader experiences of chronic illness and disability [47, 51, 52].

As observed in other social and health research contexts [53-55], diverging understandings of what constitutes illness and recovery produce challenges for designing research and health interventions. Our analysis identifies multiple health 'problems' from which participants were described as recovering, including SARS-CoV-2 viral infection, Covid-19 illness (and in some cases hospitalisation), Long Covid, or something less precise and entangled with broader social factors and the pandemic context. Processes of recovery were also diversely characterised. While a 'return to normal' was both possible and desirable for

For peer review only - http://bmjopen.bmj.com/site/about/guidelines.xhtml

some, recovery was also characterised as an unpredictable process requiring ongoing care, a process of working towards a new version of normal, or was rejected entirely.

These insights highlight the importance of interrogating the problem and goal of care and recovery when discussing Long Covid. Notions of care, patienthood, recovery, and being 'recovered' held different meanings for participants across studies, which had implications for how the experience of Long Covid became understood—for participants, their clinicians, people in their social and professional lives, and through the framing and analyses of the studies themselves. While one person's definition of being recovered from (Long) Covid might require the complete absence of symptoms or illness effects, others might feel recovered when they feel well enough to work or no longer require frequent medical interventions.

Rather than standardising clinical definitions of Long Covid [56], we propose that practical approaches to the study and care of Long Covid be developed at the intersections of emergent illness, care, and recovery experience. Our recent study of Covid care practice suggests that situating uncertain illness in this way is not only possible, but is in many ways a familiar element of adaptive healthcare practice and research that can be built upon [57].

Limitations

Qualitative research on Long Covid has emerged alongside rapidly evolving clinical understandings of Covid-19 and post-Covid illness. We have highlighted inconsistencies within and between studies regarding what counts as 'Long Covid.' There is an opportunity for comparative approaches to qualitative research that characterise how Long Covid is differently understood in relation to illness experience, care needs, and recovery expectations. We have also noted a need for greater consistency and reflection within studies regarding how they frame, design, and analyse the accounts of Long Covid they produce. Many qualitative studies included in our synthesis were developed as an adjunct to surveys, cohorts, and/or clinical studies, rather than designed as stand-alone qualitative studies. There is scope for greater theoretical, sociological, as well as critical methodological engagement in this emergent field of qualitative study. Included studies primarily draw from the UK and US and are limited to studies published in English.

Conclusion

We accentuate Long Covid as a site of multiple qualitative interpretation, giving rise to coexisting versions of illness, patienthood, and recovery. Crucially, we find that Long Covid develops meaning through its social relations of varied and diverse lived experience. We caution against attempts to align or stabilise constitutions of Long Covid illness, patienthood, and recovery in a singular trajectory or understanding. Accounts of patients' experiences illuminate how illness, patienthood, and recovery are sites of continual negotiation and thus also defining qualitative features of what Long Covid is. Efforts to diagnose and intervene must be as adaptive as the varieties of Long Covid lived experience.

For peer review only

For peer review only - http://bmjopen.bmj.com/site/about/guidelines.xhtml

Acknowledgements

We acknowledge funding support from the UK National Institute for Health and Care Research (grant NIHR135315). The Centre for Social Research in Health at the University of New South Wales (UNSW) is supported by a grant from the Australian Government Department of Health. We are grateful for support from the UNSW SHARP (Tim Rhodes) and Scientia Fellowship (Kari Lancaster) schemes. Kari Lancaster is supported by an Australian Research Council DECRA Fellowship (DE230100642). We are also grateful to the St Vincent's Hospital Sydney ADAPT Study for their partnership and collaboration.

Author contributions

MH led the conception and design of the review, data collection, and analysis. TR and KL contributed to research design and analysis. MH drafted the manuscript, with all authors contributing to write-up and revision.

Funding

We acknowledge funding support from the UK National Institute for Health and Care Research (grant NIHR135315). We are grateful for additional support from the UNSW SHARP (Tim Rhodes) and Scientia Fellowship (Kari Lancaster) schemes. Kari Lancaster is supported by an Australian Research Council DECRA Fellowship (DE230100642).

Competing interests

None declared.

Data availability statement

Not applicable.

Ethics approval

Not required—review. Ethics approval linked to grant NIHR135315 was granted via the London School of Hygiene and Tropical Medicine Observational Research Ethics Committee (Ref 28635).

Figure and table legend

Figure 1: Flow diagram of phases of sampling for qualitative interpretative synthesis, adapted from existing CIS approach [28]
Table 1: Included studies
Table 2: Illustrative participant extracts from studies
Table 3: Descriptors used in Long Covid in studies

For peer review only

For peer review only - http://bmjopen.bmj.com/site/about/guidelines.xhtml

For peer review only

For peer review only - http://bmjopen.bmj.com/site/about/guidelines.xhtml

References

1. Cabrera Martimbianco AL, Pacheco RL, Bagattini ÂM, Riera R. Frequency, signs and symptoms, and criteria adopted for long COVID-19: A systematic review. International Journal of Clinical Practice. 2021;75(10):e14357. DOI: 10.1111/ijcp.14357
2. Albright C, Limoges J, Rempel GR. Living with pulmonary sequelae of COVID-19 and the implications for clinical nursing practice: A qualitative systematised review. Journal of Clinical Nursing. 2023;0(0):1-11. DOI: 10.1111/jocn.16664
3. Macpherson K, Cooper K, Harbour J, Mahal D, Miller C, Nairn M. Experiences of living with long COVID and of accessing healthcare services: a qualitative systematic review. BMJ Open. 2022;12(1):e050979. DOI: 10.1136/bmjopen-2021-050979
4. Roth PH, Gadebusch-Bondio M. The contested meaning of "long COVID" – Patients, doctors, and the politics of subjective evidence. Social Science & Medicine. 2022;292:114619. DOI: 10.1016/j.socscimed.2021.114619
5. Barker KK, Whooley O, Madden EF, Ahrend EE, Greene RN. The long tail of COVID and the tale of long COVID: Diagnostic construction and the management of ignorance. Sociology of Health & Illness. 2022. DOI: 10.1111/1467-9566.13599
6. Greenhalgh T, Knight M, A'Court C, Buxton M, Husain L. Management of post-acute covid-19 in primary care. BMJ. 2020;370:m3026. DOI: 10.1136/bmj.m3026
7. Hunt J. Making space for disability studies within a structurally competent medical curriculum: reflections on long Covid. Medical Humanities. 2023;49(1):105-16. DOI: 10.1136/medhum-2022-012415
8. Almas T, Malik J, Alsubai AK, Jawad Zaidi SM, Iqbal R, Khan K, et al. Post-acute COVID-19 syndrome and its prolonged effects: An updated systematic review. Annals of Medicine and Surgery. 2022;80:103995. DOI: 10.1016/j.amsu.2022.103995
9. Iqbal FM, Lam K, Sounderajah V, Clarke JM, Ashrafian H, Darzi A. Characteristics and predictors of acute and chronic post-COVID syndrome: A systematic review and meta-analysis. EClinicalMedicine. 2021;36:100899. DOI: 10.1016/j.eclinm.2021.100899
10. Nguyen NN, Hoang VT, Dao TL, Dudouet P, Eldin C, Gautret P. Clinical patterns of somatic symptoms in patients suffering from post-acute long COVID: a systematic review. European Journal of Clinical Microbiology & Infectious Diseases. 2022;41(4):515-45. DOI: 10.1007/s10096-022-04417-4
11. Michelen M, Manoharan L, Elkheir N, Cheng V, Dagens A, Hastie C, et al. Characterising long COVID: a living systematic review. BMJ Global Health. 2021;6(9):e005427. DOI: 10.1136/bmjgh-2021-005427
12. Akbarialiabad H, Taghrir MH, Abdollahi A, Ghahramani N, Kumar M, Paydar S, et al. Long COVID, a comprehensive systematic scoping review. Infection. 2021;49(6):1163-86. DOI: 10.1007/s15010-021-01666-x
13. Kopanczyk R, Kumar N, Papadimos T. Post-Acute COVID-19 Syndrome for Anesthesiologists: A Narrative Review and a Pragmatic Approach to Clinical Care. Journal of Cardiothoracic and Vascular Anesthesia. 2022;36(8, Part A):2727-37. DOI: 10.1053/j.jvca.2021.09.051
14. van Kessel SAM, Olde Hartman TC, Lucassen PLBJ, van Jaarsveld CHM. Post-acute and long-COVID-19 symptoms in patients with mild diseases: a systematic review. Family Practice. 2021;39(1):159-67. DOI: 10.1093/fampra/cmab076

For peer review only

15. Wong TL, Weitzer DJ. Long COVID and Myalgic Encephalomyelitis/Chronic Fatigue Syndrome (ME/CFS)—A Systemic Review and Comparison of Clinical Presentation and Symptomatology. Medicina. 2021;57(5):418. DOI: 10.3390/medicina57050418
16. Fugazzaro S, Contri A, Esseroukh O, Kaleci S, Croci S, Massari M, et al. Rehabilitation Interventions for Post-Acute COVID-19 Syndrome: A Systematic Review. International Journal of Environmental Research and Public Health. 2022;19(9):5185. DOI: 10.3390/ijerph19095185
17. Rinn R, Gao L, Schoeneich S, Dahmen A, Anand Kumar V, Becker P, et al. Digital Interventions for Treating Post-COVID or Long-COVID Symptoms: Scoping Review. J Med Internet Res. 2023;25:e45711. DOI: 10.2196/45711
18. Yong SJ, Liu S. Proposed subtypes of post-COVID-19 syndrome (or long-COVID) and their respective potential therapies. Reviews in Medical Virology. 2022;32(4):e2315. DOI: 10.1002/rmv.2315
19. Hossain MM, Das J, Rahman F, Nesa F, Hossain P, Islam AMK, et al. Living with “long COVID”: A systematic review and meta-synthesis of qualitative evidence. PLOS ONE. 2023;18(2):e0281884. DOI: 10.1371/journal.pone.0281884
20. Zheng X, Qian M, Ye X, Zhang M, Zhan C, Li H, et al. Implications for long COVID: A systematic review and meta-aggregation of experience of patients diagnosed with COVID-19. Journal of Clinical Nursing. 2022;0(0):1-18. DOI: 10.1111/jocn.16537
21. Callard F, Perego E. How and why patients made Long Covid. Social Science & Medicine. 2021;268:113426. DOI: 10.1016/j.socscimed.2020.113426
22. Rhodes T, Lancaster K. Evidence-making interventions in health: A conceptual framing. Social Science & Medicine. 2019;238:112488. DOI: 10.1016/j.socscimed.2019.112488
23. Mol A, Hardon A. What COVID-19 may teach us about interdisciplinarity. BMJ Global Health. 2020;5(12):e004375. DOI: 10.1136/bmjgh-2020-004375
24. Dixon-Woods M, Cavers D, Agarwal S, Annandale E, Arthur A, Harvey J, et al. Conducting a critical interpretive synthesis of the literature on access to healthcare by vulnerable groups. BMC Med Res Methodol. 2006;6(1):35. DOI: 10.1186/1471-2288-6-35
25. Booth A. Harnessing Energies, Resolving Tensions: Acknowledging a Dual Heritage for Qualitative Evidence Synthesis. Qual Health Res. 2019;29(1):18-31. DOI: 10.1177/1049732318808247
26. Booth A, Carroll C, Ilott I, Low LL, Cooper K. Desperately Seeking Dissonance:Identifying the Disconfirming Case in Qualitative Evidence Synthesis. Qual Health Res. 2013;23(1):126-41. DOI: 10.1177/1049732312466295
27. Barnett-Page E, Thomas J. Methods for the synthesis of qualitative research: a critical review. BMC Med Res Methodol. 2009;9(1):59. DOI: 10.1186/1471-2288-9-59
28. Harrison M, Rhodes T, Lancaster K. How do care environments shape healthcare? A synthesis of qualitative studies among healthcare workers during the COVID-19 pandemic. BMJ Open. 2022;12(9):e063867. DOI: 10.1136/bmjopen-2022-063867
29. Greenhalgh T, Peacock R. Effectiveness and efficiency of search methods in systematic reviews of complex evidence: audit of primary sources. BMJ. 2005;331(7524):1064-5. DOI: 10.1136/bmj.38636.593461.68
30. Aghaei A, Zhang R, Taylor S, Tam CC, Yang CH, Li X, et al. Social Life of Females with Persistent COVID-19 Symptoms: A Qualitative Study. Int J Environ Res Public Health. 2022;19(15). DOI: 10.3390/ijerph19159076

For peer review only - http://bmjopen.bmj.com/site/about/guidelines.xhtml

31. Bergmans RS, Chambers-Peeple K, Yu C, Xiao LLZ, Wegryn-Jones R, Martin A, et al. 'I'm still here, I'm alive and breathing': The experience of Black Americans with long COVID. Journal of Clinical Nursing. 2023. DOI: 10.1111/jocn.16733
32. Burton A, Aughterson H, Fancourt D, Philip KEJ. Factors shaping the mental health and well-being of people experiencing persistent COVID-19 symptoms or 'long COVID': qualitative study. Bjpsych Open. 2022;8(2). DOI: 10.1192/bjo.2022.38
33. Callan C, Ladds E, Husain L, Pattinson K, Greenhalgh T. 'I can't cope with multiple inputs': a qualitative study of the lived experience of 'brain fog' after COVID-19. Bmj Open. 2022;12(2). DOI: 10.1136/bmjopen-2021-056366
34. Chasco EE, Dukes K, Jones D, Comellas AP, Hoffman RM, Garg A. Brain Fog and Fatigue following COVID-19 Infection: An Exploratory Study of Patient Experiences of Long COVID. International Journal of Environmental Research and Public Health. 2022;19(23). DOI: 10.3390/ijerph192315499
35. Cooper E, Lound A, Atchison CJ, Whitaker M, Eccles C, Cooke GS, et al. Awareness and perceptions of Long COVID among people in the REACT programme: Early insights from a pilot interview study. Plos One. 2023;18(1). DOI: 10.1371/journal.pone.0280943
36. Fang C, Baz SA, Sheard L, Carpentieri JD. 'I am just a shadow of who I used to be'—Exploring existential loss of identity among people living with chronic conditions of Long COVID. Sociol Health Illn. 2023. DOI: 10.1111/1467-9566.13690
37. Maclean A, Hunt K, Brown A, Evered JA, Dowrick A, Fokkens A, et al. Negotiation of collective and individual candidacy for long Covid healthcare in the early phases of the Covid-19 pandemic: Validated, diverted and rejected candidacy. SSM - Qualitative Research in Health. 2023;3:100207. DOI: 10.1016/j.ssmqr.2022.100207
38. Moretti C, Collaro C, Terzoni C, Colucci G, Copelli M, Sarli L, et al. Dealing with uncertainty. A qualitative study on the illness' experience in patients with Long-COVID in Italy. Acta Biomed. 2022;93(6):e2022349. DOI: 10.23750/abm.v93i6.14059
39. O'Brien KK, Brown DA, McDuff K, St Clair-Sullivan N, Solomon P, Carusone SC, et al. Conceptualising the episodic nature of disability among adults living with Long COVID: a qualitative study. Bmj Global Health. 2023;8(3). DOI: 10.1136/bmjgh-2022-011276
40. Rushforth A, Ladds E, Wieringa S, Taylor S, Husain L, Greenhalgh T. Long Covid – The illness narratives. Social Science & Medicine. 2021;286:114326. DOI: 10.1016/j.socscimed.2021.114326
41. Russell D, Spence NJ, Chase JD, Schwartz T, Tumminello CM, Bouldin E. Support amid uncertainty: Long COVID illness experiences and the role of online communities. SSM Qual Res Health. 2022;2:100177. DOI: 10.1016/j.ssmqr.2022.100177
42. Schmachtenberg T, Muller F, Kranz J, Dragaqina A, Wegener G, Konigs G, et al. How do long COVID patients perceive their current life situation and occupational perspective? Results of a qualitative interview study in Germany. Frontiers in Public Health. 2023;11. DOI: 10.3389/fpubh.2023.1155193
43. Taylor AK, Kingstone T, Briggs TA, O'Donnell CA, Atherton H, Blane DN, et al. 'Reluctant pioneer': A qualitative study of doctors' experiences as patients with long COVID. Health Expectations. 2021;24(3):833-42. DOI: 10.1111/hex.13223
44. Wurz A, Culos-Reed SN, Franklin K, DeMars J, Wrightson JG, Twomey R. "I feel like my body is broken": exploring the experiences of people living with long COVID. Quality of Life Research. 2022;31(12):3339-54. DOI: 10.1007/s11136-022-03176-1

For peer review only - http://bmjopen.bmj.com/site/about/guidelines.xhtml

45. O'Hare AM, Vig EK, Iwashyna TJ, Fox A, Taylor JS, Viglianti EM, et al. Complexity and Challenges of the Clinical Diagnosis and Management of Long COVID. Jama Network Open. 2022;5(11). DOI: 10.1001/jamanetworkopen.2022.40332
46. Mattingly C, Garro L, editors. Narrative and the Cultural Construction of Illness and Healing: University of California Press; 2001.
47. Cheshire A, Ridge D, Clark LV, White PD. Sick of the Sick Role: Narratives of What "Recovery" Means to People With CFS/ME. Qualitative Health Research. 2021;31(2):298-308. DOI: 10.1177/1049732320969395
48. Brown B, Huszar K, Chapman R. 'Betwixt and between'; liminality in recovery stories from people with myalgic encephalomyelitis (ME) or chronic fatigue syndrome (CFS). Sociology of Health & Illness. 2017;39(5):696-710. DOI: 10.1111/1467-9566.12546
49. Nettleton S. 'I just want permission to be ill': Towards a sociology of medically unexplained symptoms. Social Science & Medicine. 2006;62(5):1167-78. DOI: 10.1016/j.socscimed.2005.07.030
50. Jutel A. Sociology of diagnosis: a preliminary review. Sociology of Health & Illness. 2009;31(2):278-99. DOI: 10.1111/j.1467-9566.2008.01152.x
51. Clare E. Brilliant Imperfection: Grappling with Cure: Duke University Press; 2017.
52. Bailey CW. On the Impossible: Disability Studies, Queer Theory, and the Surviving Crip. Disability Studies Quarterly. 2019;39(4). DOI: 10.18061/dsq.v39i4.6580
53. Lancaster K, Duke K, Ritter A. Producing the 'problem of drugs': A cross national-comparison of 'recovery' discourse in two Australian and British reports. International Journal of Drug Policy. 2015;26(7):617-25. DOI: 10.1016/j.drugpo.2015.04.006
54. LaMarre A, Rice C. Recovering Uncertainty: Exploring Eating Disorder Recovery in Context. Culture, Medicine, and Psychiatry. 2021;45(4):706-26. DOI: 10.1007/s11013-020-09700-7
55. Vitellone N, Theodoropoulou L, Manchot M. Recovery as a minor practice. International Journal of Drug Policy. 2022;107:103618. DOI: 10.1016/j.drugpo.2022.103618
56. Greenhalgh T, Darbyshire J, Lee C, Ladds E, J C-S. Reducing variation in the quality of long covid care: Lessons from a national quality improvement collaborative and multi-site ethnography. Research Square [Preprint]. 2023. DOI: 10.21203/rs.3.rs-3718323/v1
57. Harrison M, Rhodes T, Lancaster K. Situating 'best practice': Making healthcare familiar and good enough in the face of unknowns. SSM - Qualitative Research in Health. 2023;4:100343. DOI: 10.1016/j.ssmqr.2023.100343

For peer review only

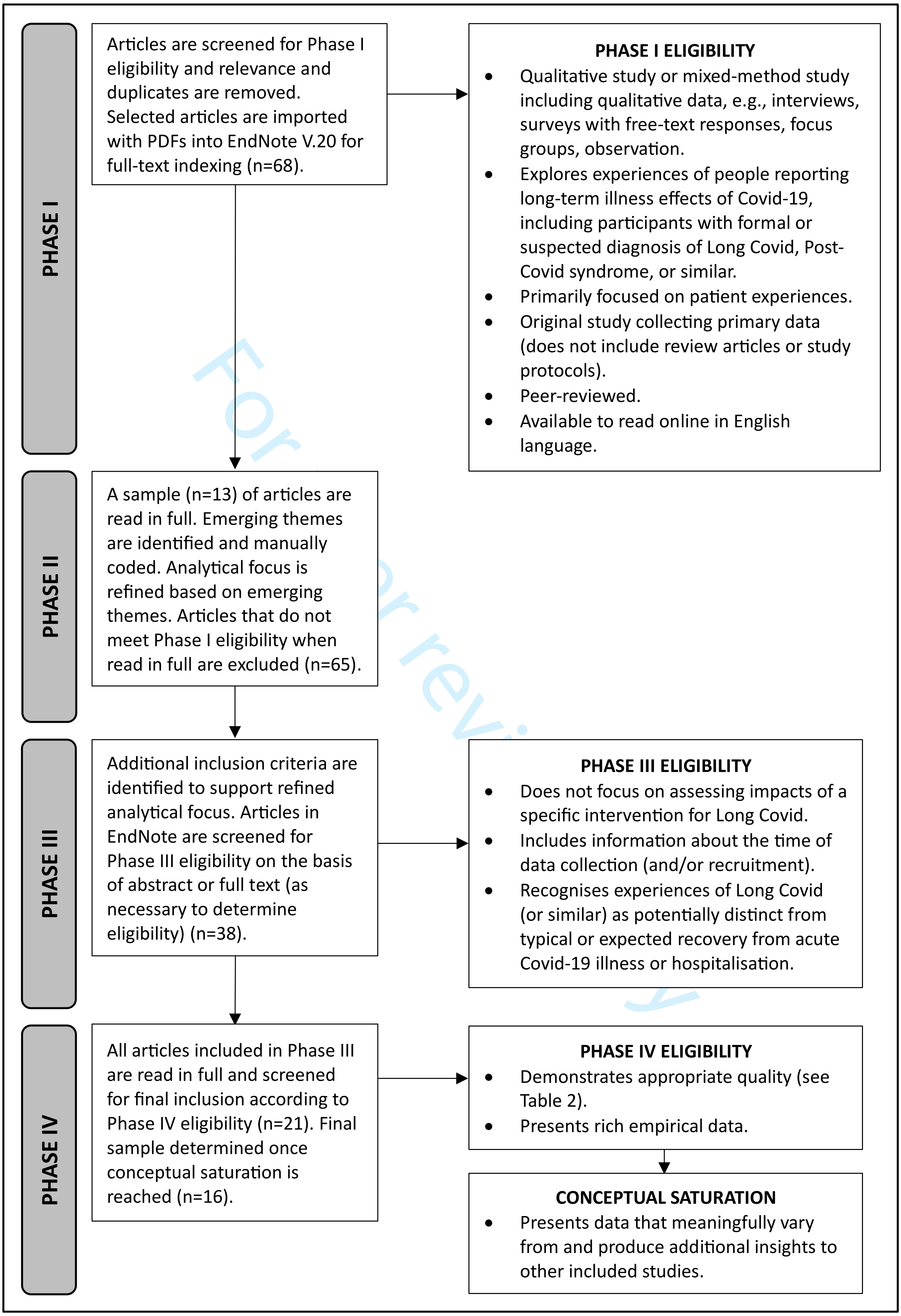
PHASE I
Articles are screened for Phase I eligibility and relevance and duplicates are removed. Selected articles are imported with PDFs into EndNote V.20 for full-text indexing (n=68).
PHASE I ELIGIBILITY
• Qualitative study or mixed-method study including qualitative data, e.g., interviews, surveys with free-text responses, focus groups, observation.
• Explores experiences of people reporting long-term illness effects of Covid-19, including participants with formal or suspected diagnosis of Long Covid, Post-Covid syndrome, or similar.
• Primarily focused on patient experiences.
• Original study collecting primary data (does not include review articles or study protocols).
• Peer-reviewed.
• Available to read online in English language.
PHASE II
A sample (n=13) of articles are read in full. Emerging themes are identified and manually coded. Analytical focus is refined based on emerging themes. Articles that do not meet Phase I eligibility when read in full are excluded (n=65).
PHASE III
Additional inclusion criteria are identified to support refined analytical focus. Articles in EndNote are screened for Phase III eligibility on the basis of abstract or full text (as necessary to determine eligibility) (n=38).
PHASE III ELIGIBILITY
• Does not focus on assessing impacts of a specific intervention for Long Covid.
• Includes information about the time of data collection (and/or recruitment).
• Recognises experiences of Long Covid (or similar) as potentially distinct from typical or expected recovery from acute Covid-19 illness or hospitalisation.
PHASE IV
All articles included in Phase III are read in full and screened for final inclusion according to Phase IV eligibility (n=21). Final sample determined once conceptual saturation is reached (n=16).
PHASE IV ELIGIBILITY
• Demonstrates appropriate quality (see Table 2).
• Presents rich empirical data.
CONCEPTUAL SATURATION
• Presents data that meaningfully vary from and produce additional insights to other included studies.

Supplemental Table 1: Database search queries

Database	Search Query	Results
PubMed	((postacute[Title/Abstract]) OR ("post acute"[Title/Abstract]) OR (pasc[Title/Abstract]) OR (persistent symptoms[Title/Abstract]) OR (prolonged symptoms[Title/Abstract]) OR ("long covid"[Title/Abstract]) OR (post covid syndrome[Title/Abstract])) AND (qualitative[Title/Abstract]) AND (covid[Title/Abstract])	144
Web of Science	(AB=(postacute) OR AB=("post acute") OR AB=(pasc) OR AB=(persistent symptoms) OR AB=(prolonged symptoms) OR AB=("long covid") OR AB=(post covid syndrome)) AND AB=(qualitative) AND AB=(covid)	187

Supplemental Table 2: Quality appraisal prompts

- Are the objectives of the research clearly stated?
- Is the research design clearly described and appropriate?
- Are data collection methods transparent?
- Are data analysis methods clearly described and appropriate?
- Are there sufficient data to support the authors' interpretations?

Quality appraisal prompts citation:

Harrison M, Rhodes T, Lancaster K. How do care environments shape healthcare? A synthesis of qualitative studies among healthcare workers during the COVID-19 pandemic. *BMJ Open*. 2022;12(9):e063867. DOI: 10.1136/bmjopen-2022-063867

For peer review only - http://bmjopen.bmj.com/site/about/guidelines.xhtml

ENTREQ Checklist

No	Item	Guide and description	Location where reported*
**1**	Aim	State the research question the synthesis addresses.	Abstract (p2); Introduction (p4, para 1–2); Methodology (p4, para 3)
**2**	Synthesis methodology	Identify the synthesis methodology or theoretical framework which underpins the synthesis, and describe the rationale for choice of methodology.	Title (p1); Abstract (p2); Introduction (p4, para 1); Methodology (p4, para 3)
**3**	Approach to searching	Indicate whether the search was pre-planned.	Methodology (p4, para 3); Search Strategy (p4, para 4); Inclusion (p4, para 5; p5, para 1); Figure 1
**4**	Inclusion criteria	Specify the inclusion/exclusion criteria.	Methodology (inclusion) (p4, para 5; p5 para 1–2); Figure 1
**5**	Data sources	Describe the information sources used and when the searches conducted; provide the rationale for using the data sources.	Abstract (p2); Methodology (search strategy) (p4, para 4); Supplemental Table 1
**6**	Electronic Search strategy	Describe the literature search.	Methodology (search strategy) (p4, para 4); Supplemental Table 1
**7**	Study screening methods	Describe the process of study screening and sifting.	Methodology (inclusion) (p4, para 5, p5, para 1–2); Figure 1
**8**	Study characteristics	Present the characteristics of the included studies.	Methodology (inclusion) (p5, para 2); Table 1 (pp5–7)

For peer review only

No	Item	Guide and description	Location where reported*
**9**	Study selection results	Identify the number of studies screened and provide reasons for study exclusion	Figure 1
**10**	Rationale for appraisal	Describe the rationale and approach used to appraise the included studies or selected findings	Methodology (inclusion) (p4, para 5; p5, para 1); Supplemental Table 2
**11**	Appraisal items	State the tools, frameworks and criteria used to appraise the studies or selected findings	Methodology (inclusion) (p4, para 5; p5, para 1); Supplemental Table 2
**12**	Appraisal process	Indicate whether the appraisal was conducted independently by more than one reviewer and if consensus was required.	Methodology (data extraction and analysis) (p7, para 1–2); Author contributions (p18, para 5)
**13**	Appraisal results	Present results of the quality assessment and indicate which articles, if any, were weighted/excluded based on the assessment and give the rationale.	Figure 1
**14**	Data extraction	Indicate which sections of the primary studies were analysed and how were the data extracted from the primary studies?	Methodology (data extraction and analysis) (p7, para 1–2); Figure 1
**15**	Software	State the computer software used, if any.	Figure 1
**16**	Number of reviewers	Identify who was involved in coding and analysis.	Methodology (data extraction and analysis) (p7, para 1–2); Author contributions (p18, para 5)
**17**	Coding	Describe the process for coding of data.	Methodology (data extraction and analysis) (p7, para 1–2)

For peer review only

No	Item	Guide and description	Location where reported*
**18**	Study comparison	Describe how were comparisons made within and across studies.	Methodology (data extraction and analysis) (p7, para 1–2)
**19**	Derivation of themes	Explain whether the process of deriving the themes or constructs was inductive or deductive.	Methodology (data extraction and analysis) (p7, para 1–2).
**20**	Quotations	Provide quotations from the primary studies to illustrate themes/constructs, and identify whether the quotations were participant quotations of the author's interpretation.	Findings (pp10–13); Table 2 (pp8–9)
**21**	Synthesis output	Present rich, compelling and useful results that go beyond a summary of the primary studies.	Findings (pp.10–13); Discussion (pp13–18); Conclusion (p18, para 3)

* Page numbers correspond with the submitted Word document version of the manuscript.

Checklist citation:

Tong, A., Flemming, K., McInnes, E. et al. Enhancing transparency in reporting the synthesis of qualitative research: ENTREQ. *BMC Med Res Methodol.* 2012;**12**(181). DOI: 10.1186/1471-2288-12-181

For peer review only
